# Mass spectrometry-based proteomics as an emerging tool in clinical laboratories

**DOI:** 10.1186/s12014-023-09424-x

**Published:** 2023-08-26

**Authors:** Alemayehu Godana Birhanu

**Affiliations:** Institute of Biotechnology, Addis Ababa University, P.O. Box 1176, Addis Ababa, Ethiopia

**Keywords:** Mass spectrometry, Clinical proteomics, Diagnosis, Biomarker, Prognosis, Microbiology, Cancer, Metabolic Disorders

## Abstract

Mass spectrometry (MS)-based proteomics have been increasingly implemented in various disciplines of laboratory medicine to identify and quantify biomolecules in a variety of biological specimens. MS-based proteomics is continuously expanding and widely applied in biomarker discovery for early detection, prognosis and markers for treatment response prediction and monitoring. Furthermore, making these advanced tests more accessible and affordable will have the greatest healthcare benefit.

This review article highlights the new paradigms MS-based clinical proteomics has created in microbiology laboratories, cancer research and diagnosis of metabolic disorders. The technique is preferred over conventional methods in disease detection and therapy monitoring for its combined advantages in multiplexing capacity, remarkable analytical specificity and sensitivity and low turnaround time.

Despite the achievements in the development and adoption of a number of MS-based clinical proteomics practices, more are expected to undergo transition from bench to bedside in the near future. The review provides insights from early trials and recent progresses (mainly covering literature from the NCBI database) in the application of proteomics in clinical laboratories.

## Introduction

Proteins are biomolecules that better bridge the gap between genomic information and biologic functions and disease phenotypes [1]. Proteins do not function in isolation and major biological processes are mediated through protein interactions that control metabolic and signaling pathways, cellular processes, and organismal systems, hence control the chaotic networks and mechanisms implicated in health and diseases [2–4]. Proteomics is an integrated research area that is centered on the premise of large-scale identification and quantification of proteins in biological specimens [1, 5]. The high sensitivity and specificity achievable by mass spectrometry (MS) make it superior to immunoassays for analysis of several drug types [6, 7]. MS-based clinical proteomics improve medical practice at the level of diagnosis, characterizing new targets for drug development, therapeutic intervention, prognosis and digging for biomarker candidates [8–14]. Current proteome research has a strong emphasis on biomarker discovery and validation to help with disease diagnosis, therapy monitoring, and prognosis [15, 16]. During the last decades, MS-based proteomics has led to the discovery and identification of thousands of potential protein biomarkers for a number of diseases [17]. Depending on the information which they provide, biomarkers can be divided into diagnostic, prognostic, and treatment predictive biomarkers [18]. A diagnostic biomarker is used for early detection of the disease. For example, decreased expression of full length amyloid beta (Aβ) peptide and increased tau protein in CSF were reported to be the only clinically validated biomarkers for Alzheimer’s disease(see (Olsson et al. (2016)) for a review of these biomarkers) [19]. A prognostic biomarker is typically utilized to foretell the recurrence and severity of disease as well as patient’s response to treatment by a given drug. Recently, a study by Jang et al. (2021) identified five proteins, HNRNPA1, LTBP4, MRPS23, POLDIP2, and WBSCR16, to be prognostic biomarkers in adrenal cortical carcinoma (ACC) [20]. Predictive biomarkers are useful tools to classify the patients into responder and non-responder groups [21], which are all important in drug design applications [22, 23]. For example, proteins FKBP4 and S100A9 were reported as potential prediction markers of therapeutic response to neoadjuvant chemotherapy in breast cancer patients [24]. Furthermore, overexpression of SHP27 was said to predict doxorubicin resistance [25]. Many diseases, including cancer, are regulated at the protein level, making the field of proteomics important. Thus, proteins are of great importance in the diagnosis and understanding of most diseases and pathological disturbances that occur, thus having an impact on biomarkers. In the last decade, the US Food and Drug Administration (FDA) has approved a number of MS-based in vitro diagnostic methods for pathogen identification, newborn screening, quantification of therapeutic drugs in the circulation, and vitamin D assay [26].

Liquid chromatography (LC) coupled to tandem MS (LC-MS/MS) is the most widely used technique for the comprehensive identification and quantification of proteins. In this technique, proteins from biological samples are isolated and enzymatically digested into peptides, most commonly with trypsin, before separation by LC and electrospray ionization to enter the mass spectrometer. Peptide identification occurs through determination of the mass to charge ratio (m/z) of precursor ions in MS1 spectra, selection, and fragmentation of precursor ions in a collision cell, then determination of the m/z of the product ions generated from collision in MS2 spectra. Finally, protein identification is inferred from analysis their respective peptide data [27, 28]. The exact identification and quantitation of proteins are essential for a better understanding of biological processes implicated in health and disease.

Precise multiplexed quantification of proteins can be achieved by targeted proteomic methods using multiple or parallel or selected reaction monitoring (MRM/PRM/SRM). MRM rely on a triple-quadrupole (QQQ) MS to allow passage and analyses of only predefined targeted proteotypic peptides, by specifically selecting precursor ions in Q1 and their specific fragment ions in Q3 as predefined mass to charge (m/z) values [29]. The signal intensities of SRM transitions (precursor/fragment ion pairs) of the unique peptide can be monitored over time and are efficient as surrogate measures of the quantity of a specific protein. The method has high repeatability, reproducibility and broad dynamic range enabling excellent absolute and relative protein quantification across multiple biological samples, especially in the area of biomarker research [30]. These targeted proteomics strategies are used both in biomarker validation and in accurate and specific quantification of several biomarkers [31]. Mermelekas et al., (2015) has presented a summary of validated urinary biomarkers in different diseases, including cancer and diabetes, using SRM/MRM assays [31]. Jones et al., (2016) has developed MRM assay and quantified 187 candidate marker proteins for colorectal cancer (CRC) [32]. Kontostathi et al., (2019) has presented a summary of studies based on MRM targeted proteomic assays to discover and validate diseases specific biomarkers in plasma samples [33]. The application of this powerful tool is limited by its relatively low throughput.

Data-dependent acquisition (DDA) and data-independent acquisition (DIA), are other two discovery platforms used in the identification and quantification of proteins [34]. DDA is the most widely used approach where quantification is achieved by combining DDA with stable isotope labeling [34]. This technique has poor reproducibility due to due to random ion sampling especially when assessing large number of samples [27, 35]. In DIA, for example, sequential windowed acquisition of all theoretical fragment ion spectra mass spectrometry (SWATH-MS), allows comprehensive detection and quantitation of virtually every detectable compound in a sample, thereby eliminating the risk of missing a critical component and overcomes some of the limitations of DDA [35–38]. The introduction of ultrafast scanning high-resolution Q-TOF instruments led to the emergence of this novel MRM-like method, SWATH-MS based label-free quantitative proteomics, which is a faster and higher throughput alternative that can detect 30,000–40,000 peptides across large sets of samples [38, 39]. Instrumental parameters such as the size of the precursor mass windows or the resolution can be modified to improve protein depth and analytical precision in a DIA-MS method [40]. Furthermore, the type of LC column and the length of separation gradient can each alter the number of peptides detected and their resolution. A study by Chang et al., (2015) reported 30 differentially expressed proteins from a label-free SWATH analysis of synaptic proteome in between Alzheimer’s disease patients and controls [41]. Kim et al., (2018) developed a targeted DIA assay and detected candidate biomarker KRAS mutations to predict therapy response [42]. Anjo et al., (2017) briefly summarized the clinical and fundamental researches based on SWATH-MS, which led to the identification of a number of candidate biomarkers for different diseases [43]. A recent review by Boys et al., (2023) presented several studies based on DIA- and DDA-MS for the discovery and validation of different biomarker categories [34].

**Fig. 1:**
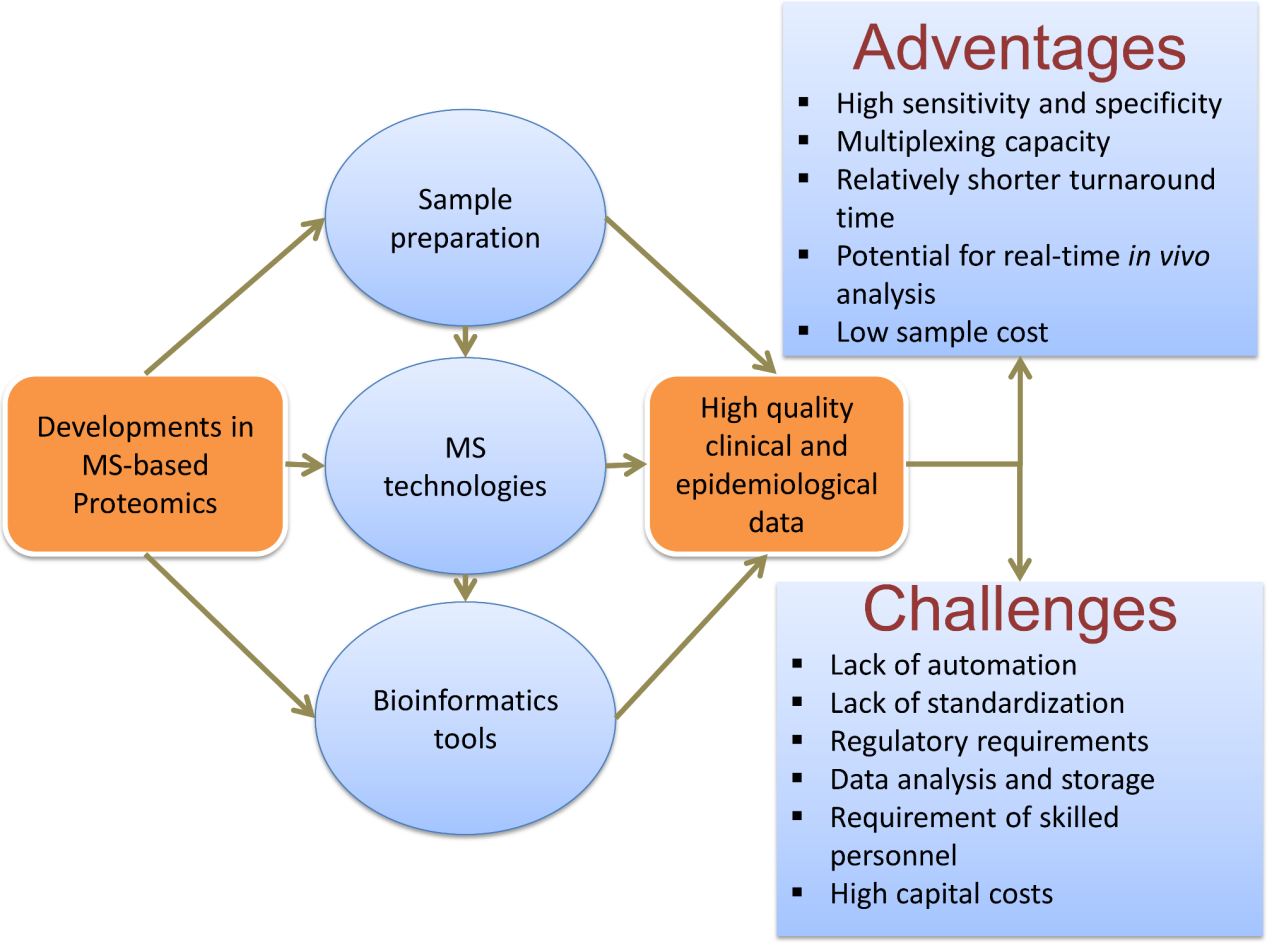
Current developments in Mass spectrometry-based clinical proteomics in sample preparation, MS technologies, bioinformatic tools, major advantages and the challenges that have to be considered

Current developments in sample preparation methods, protein quantitation strategies, MS configurations and data analysis have all been essential to address the clinical questions that advance the discovery and validation of clinically-relevant diseases biomarkers (Fig. 1) [17, 28, 44]. These progresses in MS related technologies, sample preparation methods, labeling reagents, stable isotope labeling reagents and peptide synthesis technologies, and bioinformatics have led to identification and quantification of several thousand proteins in one experiment with steadily improved sensitivity, resolution and specificity propelled proteomics into the clinic [12, 15, 45–47].

Sample preparation an important step in the proteomic characterization of clinical samples to simplify complex biological matrices (serum, urine, etc.), adjust analyte concentration(s) for the detection limit of the LC-MS/MS, exchange the sample matrix to a simpler solvent/water injection solution compatible with the LC method [28, 48]. The most commonly analyzed biofluids include blood (plasma, serum) and urine [49], expressed prostatic secretions [50], saliva [51], tears [52], cerebrospinal fluid (CSF) [53], and ascites [54, 55]. Rigorous and purpose-designed standard operating procedures are required for the diverse types of clinical samples in a non-invasive or minimally invasive manner (i.e. liquid biopsies) [28, 56]. Furthermore, sample preparation protocols in MS-based clinical laboratories have to deliver the desired cost/reportable test, practicality and robustness [48]. Antibodies are often used in targeted proteomics to quantify low abundant peptides (on the order of pg/mL in blood) [57]. For clinical applications, sample preparation and LC-MS has to be integrated into the system [47]. One of the barriers in implementing MS in clinical laboratory has been the challenge of interfacing MS and automated sample preparation instruments to laboratory information systems (LIS) for electronic order and result transmission.

Formalin-fixed paraffin-embedded (FFPE) tissue represents the gold standard non-liquid matrix in clinical analysis, enabling the long-term storage of samples and the generation of large tissue banks [58, 59]. The formation of amine–thiol cross-linking and methylene bridges inactivate enzymatic activity, thereby stabilize the biomolecules within the tissue. Mass spectrometry-based proteomics relies on protein digestion and peptide purification. The application of this technique on FFPE tissues requires reversal of chemical cross-linking and the removal of reagents that are incompatible with mass spectrometry using the appropriate protocol [60]. There is no a universal sample preparation protocol and the type of method should be optimized/selected based on the sample type and complexity, and the goal of the study [28]. Different cocktails of reagents including calibrators, quality control samples and ready-to-use solvents are now commercially available for the cell lysis, protein extraction and solubilization from clinical samples, as revised in different papers [28, 61–63].

Electrospray ionization (ESI) continues to rely on well-established reversed-phase nano-LC technologies, or combined with capillary electrophoresis [64]. Orthogonal peptide separation techniques using reverse-phase LC and strong cation exchange chromatography have grown in popularity amongst clinical proteomics research. Fractionation of peptide pool using this approach has been reported to increased proteome coverage over a single-shot experiment by up to 39% in lung cancer cell lines [65]. Stone, L. 2017, has described the different LC-MS/MS sample preparation types used in clinical laboratory [48]. Ion mobility based separation of gas phase peptide ions, as in high-field asymmetric ion mobility spectrometry (FAIMS) and trapped ion mobility spectrometry (TIMS), have been shown to reduce MS1 complexity and MS2 contamination from co-eluted and co-isolated peptides [66, 67], thereby increasing peptide detection by 30% [68].

Some of the recent developments in clinical proteomics sample preparation include filter aided sample preparation (FASP) [69, 70], MStern [71], suspension trapping (S-trap) [72, 73], the solid-phase-enhanced sample preparation (SP3) [74–77] and the in-StageTip (iST) [78]. Comparative studies have been performed to see the performance between the above listed methods [79, 80]. Compared to in-solution digestion, the FASP-based methodology is efficient to remove contaminating small molecules and salts including SDS prior to mass spectrometry analysis [79, 80]. Besides, FASP requires relatively high salt concentration for elution of the tryptic peptides, which requires a desalting procedure before MS analysis. Furthermore these additional handling steps are potentially associated with peptide losses [71]. The S-Traps method provide the most efficient protein digestion and identification in a shorter time when compared to FASP and in-solution digestions [79]. The in-StageTip-based sample processing is performed in a single, enclosed volume, which eliminates the potential of contamination and sample loss [78]. The method based on MStern utilizes the strengths of filter-based sample preparation methods and overcomes their limitation, without the compromising the protein identification in improved throughput [71]. The SP3 protocol uses a hydrophilic interaction mechanism for removal of unwanted sample components. It is reported to be simple and efficient protocol to analyze simple and complex protein mixtures in large and very small amounts, which can be easily completed within ~ 30 min [81].

Further developments in the MS equipment configuration have propelled clinical proteomics to the next level, thereby hasten the transition of MS from analytical chemistry laboratories to the clinics. This is achieved mainly due to the development of high- and ultrahigh resolution accurate-mass (HRAM) spectrometers, such as time of flight mass spectrometry (TOF MS), Fourier-transform ion cyclotron resonance MS (FT-ICR MS), and Orbitrap (Orbital ion-trap) [82–87]. The major enhancements in HRAM are made to the ion source, ion transmission, instrument tuning (for sensitivity improvements), detector adjustments, ion optics, electronics, and detector acquisition speed (for increased resolution). Advanced ionization process, operating system, and the required reagents including calibrators, quality control samples and ready-to-use solvents are now commercially available. Several ionization methods, such as desorption electrospray ionization (DESI) [88], probe electro spray ionization (PESI) [89] and rapid evaporative ionization mass spectrometry (REIMS) [90] have been used for intraoperative rapid pathological diagnosis and real-time analysis of biological samples [47]. Overall, a number of achievements are made in MS fragmentation and detection techniques and MS scanning modes as described in Macklin et al., (2020) [28].

For example, ion mobility MS combines the separation of ionized molecules based on their mobility in a carrier buffer gas, with the high-accuracy resolving power of HRAM. In trapped ion mobility, the ions are MS trapped during ion mobility separation, which allows for sequential fragmentation over a series of timed millisecond scans. Combining trapped ion mobility with parallel accumulation-serial fragmentation (PASEF) enables the selection and fragmentation of multiple precursors in a single 50 ms run, resulting in thousands of protein identifications over a short run time using nanogram amounts of material without a decrease in sensitivity, ideally suited for complex, high-throughput proteomics. A recent advancement has been made in trapped ion mobility MS that combines time-of-flight and trapped ion mobility MS (timsTOF) with liquid chromatography and improved automation software. Some of the recent achievements are also described below under chapter “Mass Spectrometry-based Clinical Pathology”.

The analysis of large and complex/heterogeneous biological data generated from MS experiments require the development of computational tools (new software and algorithms) to analyze and statistically evaluate data. In recent years there are also developments in tools and methods used to process the raw mass spectral data, including global and targeted identification and quantification of peptides and proteins. For example, a team from the Max Planck Institute of Biochemistry released a new version of the pioneering and widely used MaxQuant software platform, MaxQuant 2.0., for analyzing and interpreting data produced from MS-based proteomics research [91]. Chen et al., (2020) revised a number of recently developed bioinformatics tools used in MS-based proteomics data analysis [92].

MS-based proteomics is implemented in clinics to understand the pathophysiology of several diseases that include infectious diseases, antimicrobial susceptibility testing, phylogenetic classification, urine toxicology screening, new-born metabolic screening, clinical metabolic profiling and non-communicable pathological conditions such as cancer, metabolic disorders, amyloidosis, disorders of the immune system, and characterization of renal diseases, reproductive diseases, blood disorders and ocular diseases [46, 93, 94]. The main purpose of the manuscript is to discuss the major achievements, challenges and future prospects in MS-based clinical proteomics. It covers some of the recent technologies in clinical pathology, like MSI and emerging in vivo techniques, and applications related to communicable diseases caused by microbial pathogens (bacterial, viral and fungal diseases) and non-communicable pathological conditions (cancer and metabolic disorders).

## Biomarker discovery workflows

Biomarker research follows a continuum that begins with the putative biomarker discovery, and proceeds through candidate prioritization, verification and validation to the eventual clinical application and post-implementation monitoring. The discovery phase requires high confidence identification and simultaneous quantification of biomarker candidates, which gives clues about proteins that shows statistical significant changes in response to a given environmental change, drug treatment. Identification of medium to low abundance proteins without enrichment from complex biological samples is one of the biggest challenges in biomarker discovery [95]. The discovery phase generates 100 to 1000 s of candidates and the candidates that show significant differences between cases and controls have to be prioritized. Furthermore, proteins secreted or on the cell surface, acting in known cellular pathways or hypothesized to be deregulated in the diseased state are targeted for testing [96].

Alternatively, targeted proteomics approaches like the multiple reaction monitoring (MRM) MS/MS multiplexed assays and Stable Isotope Standard with Capture by Anti-Peptide Antibodies (SISCAPA) can be used to prioritize selected biomarker candidates for validation [97–99]. The identified and prioritized candidate biomarkers have to be validated in a larger sample size covering a broad section of patient cohorts. A high throughput workflow with high specificity and sensitivity is employed in verification phase to confirm the identification and screen only fewer but higher quality leads into the costly validation phase. The validation phase assesses the biomarker performance characteristics in real clinical practice, and determines the range of conditions under which the biomarker will deliver high-quality, reliable and reproducible research data necessary for the effective use of biomarkers [96]. The MS-based biomarker research and development approach may solve several vexing issues with the conventional immunoassays; accuracy, selectivity, specificity and multiplexing (MS can measure > 100 peptides at a very little incremental cost per added analyte) [100, 101]. Besides, the MS-based approach performs a direct measurement of analytes with wide dynamic range [101].

Recently, a fully automated, clinically validated HPLC-MS/MS in MRM mode has been reported for identification and quantification of wild-type and variant amyloid β (Aβ) peptides in cerebrospinal fluid of alzheimer’s disease (AD) [102]. Examples of target analytes and clinical areas are summarized by van der Gugten, J. Grace, (2020) [101]. Chambers et al., (2014) and N. Leigh Anderson (2010) have presented a list of FDA approved or cleared cancer biomarkers based on targeted proteomics [100, 103]. Some of the FDA approved MS-based protein biomarkers are shown in Table 1 below.

**Table 1:** Examples of LC-MS/MS- based FDA approved tests in clinical laboratory

Technology	Identification	Sample	Diseases	Year	Ref.
HPLC-MS/MS (MRM)	Wild-type (wt) amyloid β (Aβ)	Cerebrospinal fluid / FFPE	Alzheimer’s disease (AD)	2020	[102]
VITEK-MS (VITEK 2 )	Microbes and AMR profile	Microbial sample	Infectious diseases	2013	[104]
Bruker MALDI Biotyper CA System	Gram-negative and gram-positive bacteria and yeast	Bacterial sample	Infectious diseases	2013	[105, 106]
OVA1, in vitro diagnostic multivariate index assay (SELDI-TOF-MS)	CA 125, TTR, ApoA1, β-2 microglobulin, TF	Serum	Ovarian cancer	2009	[107]

Clinical proteomics has the ability to delineate the functional units of a cell, more likely driving the phenotypic differences of a disease. Despite the recent advances in the area of technology development/standardization and bioinformatics to enable confident identification of molecular disease signatures, major roadblocks have been impeding an efficient transition of protein candidates in to clinical biomarker with only few biomarkers have been approved by the FDA over the last two decades [108]. This is in contrast with over a thousand claimed biomarker candidates reported in scientific literature for cancer alone, indicating a discrepancy between discovery and validation. Several resources are available for determining development processes and acceptability criteria for specific LC–MS/MS assays, and many of them are general recommendations or are specific to research applications that may not translate clinically [109].

Several barriers attributable to this discrepancy has been identified, including a lack of high quality, well-annotated biospecimens, measurement inconsistency and a lack of reproducibility within and across proteomic platforms (analytical challenges), difficulty in verifying biomarker candidates before large-scale clinical trials using immunoassays, uncertainty of how to successfully develop and validate a method that meets guidelines required by the regulatory agency, lack of publicly accessible, high-quality affinity reagents, reference materials, and data sets for data mining, hypotheses generation, and experimental validation prior to clinical validation, lack of standardized data analysis, instrumentation/automation challenges, standardization and harmonization of MS methods and visualization tools and lack of appropriate statistical and experimental study design [108, 109]. An improved understanding of the challenges and strategies in each stage of the pipeline is fundamental for accelerating the pace of biomarker development and facilitating the implementation of novel clinical tests. Successful application of proteomics in clinics requires implementation of standards and metrics to ensure that observed changes are reflective of true disease biology, followed by proper large-scale validation.

## Application of mass spectrometry-based clinical proteomics

### Comparative proteomics in medical research

Most clinical proteomic studies rely on determination of differences in relative protein abundance in two or more conditions in a quantitative or qualitative manner [110–112]. For example, in expression proteomics-based biomarker discovery, biomarkers are detected through comparison of protein expression profile between normal samples vs. disease samples like body fluids and tumor tissues [21]. In general, comparative proteomics aims to analyze proteome changes in response to development, disease, or environment in a two-step process involving protein fractionation and protein identification by mass spectrometry [113]. The choice of MS instrument varies depending on the clinical application. For example, triple quadrupole mass spectrometers coupled to liquid chromatography are often used for quantitative analysis of most of the small molecules for newborn screening, therapeutic drug monitoring, vitamin D, and steroid assays, while matrix-assisted laser desorption/ionization (MALDI) combined with time-of-flight (TOF) mass analyzer is generally used for clinical microbiology and [26, 114]. Furthermore, SRM and PRM performed on high-resolution hybrid quadrupole-Orbitrap (Q-OT) or time-of-flight instruments are routinely used for targeted quantification of proteins in a complex biological matrix [115, 116].

### Mass spectrometry-based clinical pathology

More recently, mass spectrometry-based assays are becoming more popular in clinical diagnostic laboratories and have emerged as a promising tool for modern pathology [46]. Technological advancements that aid its practicality in pathology and clinical diagnostics include the cocktail of variations in the mass spectrometer configurations, for example, as in mass spectrometry imaging (MSI), emerging in vivo techniques, paper spray ionization mass spectrometry (PSI-MS) and MS miniaturization [117, 118]. In contrast with other established analytical assays, mass spectrometry-based assays offers high analytical specificity and sensitivity, improved diagnostic accuracy, low sample cost and multiplexing opportunities as it can identify and quantify multiple analytes in a high-throughput manner from complex samples, such as pathology specimens [46, 119–121]. Furthermore, it analyzes tissues directly without the need of time-consuming multiple staining and microscopy steps; significantly reducing the time to diagnosis or even it can be used to guide intraoperative tumor excision [121]. Furthermore, significant progresses have been made in the use of MS-based clinical pathology for the identification and confirmation of localization of renal protein deposits, thereby, help in diagnosing of amyloidosis and characterization of renal diseases [46].

#### Mass spectrometry imaging (MSI)

Mass spectrometry imaging (MSI) is an emerging analytical technique that has revolutionized biomedical and pharmacological investigations and allows simultaneous detection and visualization of the spatial distribution of biomolecules across the tissue specimens in a label-free untargeted manner for multiplex analysis [26, 122]. Desorption Electrospray Ionization (DESI) and MALDI are the most common ionization techniques used in MSI [123–125]. MSI combines the advantages of microscopic techniques and discovery-based approaches while enabling spatiotemporal analysis of complex biological samples with multiplex detection [126]. Imaging experiments are carried out by first scanning the tissue surface in 2D, then recording the mass spectral data pixel-by-pixel, which are then plotted to create the ion images [26].

The results can be displayed as single or multiple ion images producing molecular histology-like datasets. The information gained from MS and visualization of spatial distributions in thin sample sections makes MSI a valuable tool for biological specimen characterization, which provides a better understanding of the molecular basis and mechanism of diseases with relation to tissue morphology [46, 122]. Today, the scientific community uses a variety of MSI methods to investigate the distribution of proteins, peptides, and small-molecule metabolites in various biological models.

Today MSI has emerged as a valuable tool with several clinical applications in the context of disease characterization, drug development, biomarker discovery, diagnosis, and prognosis [125–128]. For instance, MSI can be used in biomarker discovery, to determine the location of the biomarker in the tissue section for differentiating between cancer and healthy specimens, tumour typing and disease staging, assessing tumor margins from excision biopsies, intraoperative tumour excision, drug localization, potential therapeutic targets, therapy prediction and diagnosing a number of other diseases [46, 123, 124, 129–133]. Thus, it is evident that MSI is making a stronger impact on the clinical decision-making process. The majority of human studies employing MSI focused on cancer and the effectiveness of this technology in other diseases (renal, infectious, skin, fertility, transplantation, and metabolic diseases) needs to be assessed in the future [26, 123].

Until recently, MSI in cancer had been performed exclusively on fresh frozen tissues. It was believed that proteins were inaccessible from the FFPE tissues. Optimized protocols has been developed to overcome the limitations and equal number of proteins can be identified from both fresh frozen and FFPE tissues, with each sample type having unique advantages and limitations as revised [127, 134, 135]. Proteolytic digestion can be done directly on fresh frozen samples, without the need for prior retrieval steps. However, fresh samples require rapid freezing to inhibit endogenous enzymatic degradation. It also requires additional careful cleanup steps to remove other biomolecules that may interrupt the detection of peptides. Using the FFPE samples, on the other hand, very large sample banks can be developed and stored at room temperature for indefinite periods of time without loss of morphological information. The challenge with FFPE samples is formation of methylene bridges and protein cross-linking caused by the formalin fixation, which makes proteins inaccessible to proteolytic digestion and must be reversed prior to further preparation steps using the widely used heat-induced antigen retrieval method.

The current state and further challenges of routinely implementing MSI in the clinical pathology laboratory are presented by experts in the field [133, 136]. Overall, MSI has high sensitivity, chemical specificity, fairly high spatial resolution, and the ability to detect multiplex molecular information. This makes MSI a powerful medical imaging tool that could be useful as an adjunct to histology for disease diagnosis [26].

#### Emerging in vivo techniques

Current MSI-based methods requires sample preparation steps, have relatively higher turnaround time and limited application in the assessment of processed tissue specimens. For the proper implementation of MSI for routine clinical use, there is a technological demand for direct, real-time and rapid analysis of unprocessed samples at atmospheric pressure with a simpler sample preparation, simpler instrumentation. These improvements made the MSI-based in vivo analysis as an attractive and feasible choice. More than 40 ambient ionization techniques, including Rapid evaporative ionization MS (REIMS), have now been described in the literature since the last decades [117, 133, 137]. REIMS was originally developed and integrated to routine clinical use for accurate identification of tumor tissues during surgery [138–141]. A number of REIMS-based non-destructive methods have been developed with high sensitivity and specificity to analyze and identify tissue samples in vivo and ex vivo without sample preparation and in real time [46, 142]. These include REIMS-associated endoscopy (iEndoscope), MasSpec Pen and the intelligent knife (iKnife) [46, 117, 139, 140, 142, 143].

### Mass spectrometry-based proteomics in diseases diagnosis

#### Diagnosis of infectious diseases

In the last decades, the field of microbiology has benefited from continued technological advances in MS and proteomics-based technologies which are increasingly used to characterize the molecular details of pathogen-host interactions and provide insights into the biological basis of infectious diseases [144–147]. MS-based clinical proteomics-based proteomics have been used for rapid identification and typing of viral, bacterial and fungal pathogens [4, 148–150]. In the microbiology laboratory, the development of MALDI-TOF allowed for the rapid microbial identification and strain typing, epidemiological studies, detection of biological warfare agents, detection of water- and food-borne pathogens, and detection of antibiotic resistance [4, 121, 148, 151, 152]. Furthermore, MS could be combined with machine learning algorithms to provide surveillance of airborne pathogens.

**Fig. 2:**
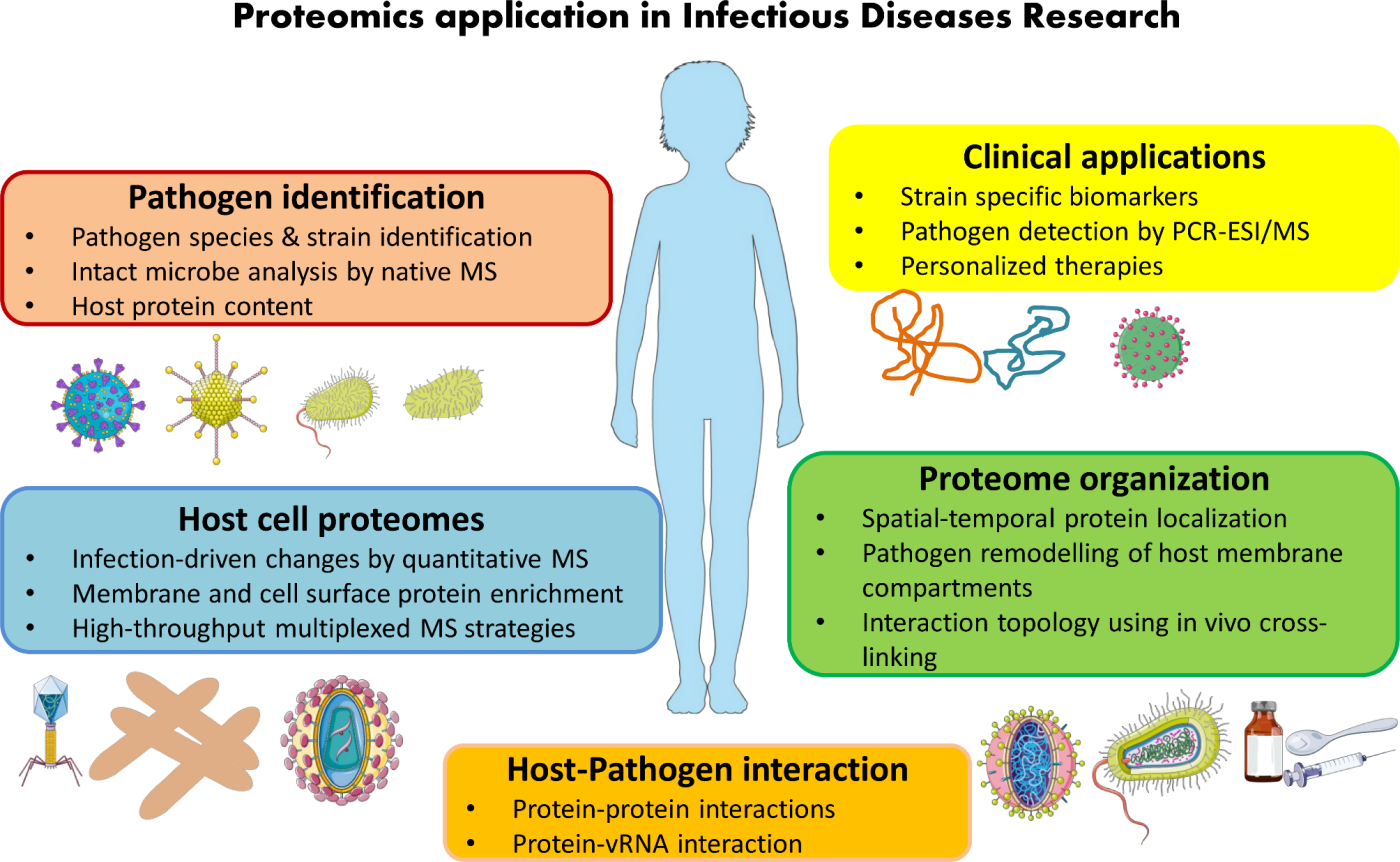
Overview of proteomic strategies and their application in infectious diseases research, modified from Greco, T. M., & Cristea, I. M. (2017)[144]

Proteomics has immense potential in characterizing protein profiles of pathogens to find a deeper knowledge of dysregulations in infection disorders, bacterial resistance and virulence – and monitoring the emergence and spread of microbial pathogens, achieve a deeper insight into pathogenesis, develop therapeutic techniques and identify new targets for future drugs [4, 153–158]. In 1996, Holland et al. showed the first MS-based protein profiles from whole cell bacteria that could be used to differentiate various species [159, 160]. Automated, standardized protocols and software packages for the analysis of bacterial MS data are available, which can be easily adapted by microbiology laboratories using either academic or commercial protocols [149, 161]. Database upgrades and sample enrichment are essential elements to refine the MS-based proteomics and increase its power [162]. For example, the company claimed that MALDI Biotyper CA System can identify 210 species or species groups, covering a library of 280 clinically important bacteria and yeast species, and representing more than 98% of the typical bacterial identification workflow of clinical microbiology laboratories. The applications of proteomics in clinical laboratory setting is summarized in Fig. 2, modified from Greco, T. M., & Cristea, I. M. (2017) [144].

#### Diagnosis of bacterial infections

One of the analytical challenges in clinical microbiology is rapid, unambiguous identification of microorganisms and the distinction from closely related species [163]. As a result, frequently additional methods are required to verify a tentative identification. MS-based methods relay on sequence-based identification, where identification of the species is supported by multiple discriminative peptide sequences that allows unambiguous identification of all infectious agents in the database used to interpret the obtained MS data [163, 164]. The microbial identification by MS has revolutionized the way the pathogenic microbes are identified from the culture of human specimens [165]. This device intercepts characteristic proteome fingerprints of a pathogen and matches them against the reference library spectra for identification with a comparable accuracy to that of a nucleic acid sequencing methods, but with better speed, easy operation, robustness, and cost-effectiveness [26, 165].

MS-based proteomic techniques have identified two secreted serodiagnostic antigens, in vitro by common *Mycobacterium tuberculosis* clinical isolates, which are potential candidates for a kit-based serum screening test [121, 166]. Many studies have demonstrated that MS-based proteomics can detect blood stream infections in shorter time and better accuracy that the conventional diagnostic methods [104, 167–171]. Guembe et al. (2014) has also reported that MALDI-TOF MS can perform better than conventional culture methods in diagnosis of catheter-related bloodstream infections [172]. Furthermore, liquid chromatography coupled to tandem MS (LC-MS/MS) is applied in a multitude of important diagnostic niches of laboratory medicine [163, 164, 171, 173, 174]. It has been shown that diagnostics based on this method required minimal processing time and identified multiple uropathogens from urine samples [173–179]. Culture based diagnosis of infectious diarrhea in laboratory is a costly and time-consuming process requiring 3–5 days for detection and identification of enteric bacterial pathogens in the stool samples. Diagnosis of infectious diarrhea based on MS shorten the turnaround time of the test to 30 min [180]. Early diagnosis is critically important in diseases like bacterial meningitis, which is a neurological emergency. MALDI-TOF MS has been used for direct detection of the bacteria causing meningitis in cerebrospinal fluids [181–184]. Singhal et al., (2015) has presented the list of bacterial pathogens in which MALDI-TOF MS was effectively used for identification and strain typing [148]. The clinical application of MALDI-TOF MS in china was first approved in 2012 [148, 185]. The two FDA-approved systems, Vitek MS and MALDI Biotyper, are proven to deliver rapid, accurate, automated, high throughput and cost-effective identification of bacteria and yeast with a library size of 572 and 406 strains, respectively [148, 186, 187].

Rapid diagnosis antimicrobial resistance is critical for the selection of optimal antibiotic treatments and better outcome of infection. MS-based proteomic have been proposed for rapid detection of antimicrobial resistance [188, 189]. Florio et al., (2020) has reviewed several MALDI-TOF MS-based methods that have been proposed for rapid detection of antimicrobial resistance [189]. Aleshukina et al., (2022) has identified promising markers of resistance for *Pseudomonas aeruginosa* and *Staphylococcus aureus* using a MALDI-TOF MS-based approach [190]. Furthermore, Weis et al., (2022) has shown the applicability of MALDI-TOF mass spectra-based machine learning approach in predicting antimicrobial resistance in clinically important pathogens [191]. Finally, Charretier & Schrenzel have discussed the practicality of MS to identify antimicrobial resistance mediated by horizontal gene transfers or by mutations that affect the quantity of a gene product, and the challenges to identify resistance mediated by target mutations in bacterial pathogens [192].

#### Diagnosis of viral infections

The use of MS in virology is still limited due to the relatively low protein content of viruses, higher molecular weight of viral proteins and a probable carryover of debris of the cell substrate in which viruses are cultured in vitro [193]. Despite the challenges, MALDI-TOF MS has been used in virology for viral identification and genotyping, subtyping, and epidemiological studies [148]. For example, several studies have proved the potential of the technique in the diagnosis of various viral pathogens like influenza viruses, enteroviruses, human papilloma viruses (HPVs), herpes virus, and hepatitis virus with a better sensitivity and lower limit of detection [194–198]. Proteomic analysis of sera from patients with severe acute respiratory syndrome (SARS) has identified higher concentrations of truncated forms of Alpha-1 antitrypsin in the sera of SARS patients compared with healthy controls [199]. Some MS platforms have been shown to detect 60 HBV variants with accuracy and low detection limits [200]. Later, Peng et al., (2013) and Calderaro et al., (2014) have proved the application of the technique in identification and typing of human enteroviruses associated with hand, foot and mouth diseases and poliovirus, respectively [201–203].

MS-based proteomics has also been used for studying emerging and re-emerging viral infections like HIV-1, CCHFV, ZIKV, and DENV, coronaviruses (MERS-CoV and SARS-CoV) as well as the recent SARS-CoV-2 pandemic [156]. Recent studies have shown the effectiveness of MS-based of protein biomarkers with the aid of machine learning algorithms for the diagnosis of SARS-CoV-2 with an accuracy of 93.9% [117, 204–207]. The development of direct sampling ionization MS using paper spray systems and robotic handler minimize the time spent for sample preparation steps to allow rapid diagnosis of hundreds of SARS-CoV-2 samples with in a day in large clinical sample cohorts [208, 209]. Several academic laboratories and industrial partners have established a Cov-MS consortium to translate the technology from analytical laboratories to clinics [210]. The Cov²MS assay was developed to diagnose SARS-CoV-2 nasopharyngeal swabs, saliva and blood plasma with a higher sensitivity and proved to be reproducible across different laboratories [211, 212]. The assay can be used to monitor dozen pathogens in pooled patient samples for early warning system for impending epidemics and pandemics and subsequent rapid development of vaccines and diagnostics [212, 213].

This will provide essential insights in investigation of disease pathogenesis and markers which may serve as potential diagnostic tools, antiviral drug and vaccine targets [156, 214, 215]. Finally, some studies have been conducted and proved the efficacy of MS-based proteomics to be a sensitive and rapid method for the detection of drug resistance against antivirals, for example, resistance to ganciclovir in cytomegaloviruses [216].

#### Diagnosis of fungal infections

The molecular diagnostic methods based on 18 S rRNA and the internal transcribed spacer regions 1 and/or 2 (ITS 1/2) are labor-intensive and time-consuming [217, 218]. MS-based fungal identification has moved at a slower pace than in bacteria, due to their inherent biological complexity and co-existence of different fungal phenotypes in the same organism [219]. Furthermore, fungal proteomics require standardized protocols/parameters like culture media, quantity/type of colony material and incubation time and cells might require additional treatment along with beating with beads to disrupt strong cell walls [148, 220]. Limited work has been reported on the use of MALDI-TOF MS in fungal strain typing or to determine antifungal drug susceptibility [218].

MS-based clinical proteomics is proved to be a rapid and accurate technique for the identification of both diagnostic biomarkers for fungal infections and therapeutic targets, strain typing, taxonomy and to determine antifungal drug susceptibility [148, 218, 221–223]. Amiri-Eliasi and Fenselau (2001) have reported the first application of the technique for identification and characterization of single-celled fungus, *Saccharomyces cerevisiae* [224]. Later, several researchers have reported MS-based proteomics to be a reliable and time-saving approach for identification of various yeast species in bloodstream infections [225–227], and in detection of various human fungal pathogens, as revised in Singhal et al., (2015) [148]. Direct MALDI TOF- MS analysis of aliquots from positive blood cultures allowed rapid and accurate identification of the main Candida species, thus obviating the need for sub-culturing on specific media [228]. Recent studies have optimized MALDI-TOF-based proteomics to identify filamentous fungi at the species level, provided that an appropriate database is available [229–234]. The protocols have been optimized for routine diagnosis of filamentous fungi and dermatophytes only after 2010 [230–233, 235–241]. Commercial products available in Europe and America for routine diagnosis of fungal infections include, Saramis, Vitek MS, the Andromas MS, and the Bruker MALDI Biotyper. The performance of each technique is tested by several publications, as reviewed by Normand et al., (2017) [106, 229].

Prediction of antimycotic resistance in fungi by MS has not advanced as much as it has, in predicting resistance bacteria, might be due to absence of drug degrading enzymes [148]. A few species Candida (*C. glabrata* or *C. krusei* and *C. parapsilosisis*) have been reported to be intrinsically resistant to azoles and echinocandins respectively [242]. Species-specific resistance has been reported in many molds and zygomycetes [243, 244]. Therefore, antimycotic resistance in fungi may be predicted simply by identification of the inflicting fungal species by MALDI-TOF MS [242].

#### Mass spectrometry based proteomics in cancer diagnosis

Cancer is the second leading cause of death after cardiovascular disease and poses a major problem to healthcare systems globally [245–247]. Current research focuses on the biomarker discovery and validation to enhance early detection, discovery of biological pathways, integrations with available genomics/transcriptomics profiles, appropriate classification of risk groups, treatment selection, therapy monitoring and prognosis in oncology clinic, which resulted in a gradual reduction in cancer mortality rates [15, 28, 245–247]. Despite the major achievements of genomic studies in cancer prognostics, treatment and diagnostics, it only provides a static image in the process of carcinogenesis [14]. Clinical research based on genomics and transcriptomics have identified numerous cancer-driving genes. The major limitation of transcriptomics dataset is that molecular differences between cancer cases and healthy controls or different stages of cancers is positively, but weakly, correlated with protein expression, which makes it difficult to directly translate to our understanding of disease biology [28, 62, 248–250]. On the other hand, proteins are ideal predictors of disease progression as they are directly involved in most biological processes and also the active targets of most cancer therapeutics [28, 251, 252]. This discordance arises due to the high dynamic and complex nature of proteome regulation [28]. For example, protein expression is affected by alternative splicing, SNP’s, transcript degradation, protein-protein interactions and degradation rates and post-translational modifications (PTMs) and requires an accurate detection technique to be used in clinical setting [28, 253, 254].

The diversity in cancer subtypes and their metastatic potential in progression of malignant cancers pose a challenge in the development of successful therapeutics [255–257]. For example, several studies have shown over 30 different of ovarian cancer subtypes, each of which arise from a different cell and has its own unique proteome [258–260]. This makes cancer diagnosis and prognosis beyond the scope of microscopic examination [261].

Proteomics has been introduced more than a decade ago to study more the dynamic molecular entities involved in cancer development and to reveal novel biomarkers of diseases. Most studies in oncoproteomics field focused on protein expression profiling across different biological groups with the aim to identify biomarkers that can be used for detection, stratification or prognosis of cancer and cancer therapies [14]. According to National Cancer Institute (NCI), a biomarker is a biological molecule found in blood, other body fluids, or tissues that provides an indication of a normal or abnormal physiological process, or the state of disease [28]. For example, during cancer progression, qualitative and quantitative changes in protein profiles occur both in tissues, blood and other body fluids [262]. Thus, clinical proteomics may provide the most accurate reflection of the tumour’s physiological state [28]. Despite the little impact of oncoproteomics on patient management and clinical decision-making to date, the search for cancer-related biomarkers with proteomics has major potential to improve risk assessment, early detection/diagnosis, prognosis, pharmacodynamics, recurrence and prediction - treatment selection and monitoring [14].

A collaborative research by the National Cancer Institute’s Clinical Proteomic Tumor Analysis Consortium (CPTAC) performed an integrated large-scale proteogenomic analysis to understand the molecular basis of different cancer types, including colorectal, breast and ovarian cancer [263]. The milestones and several NCI-sponsored research outputs from CPTAC participating partner institutions between 2009 and 2021 are published in their website https://proteomics.cancer.gov/resources/milestones-and-publications. The Proteomics Standards Initiative from the Human Proteome Organization (HUPO-PSI) has released recommendations concerning minimal information about a proteomics experiment to increase independent reproducibility of published data and the NCBI has taken a lead role in this standardization process [15, 264, 265]. Furthermore, the Early Detection Research Network was established for streamlined discovery and evaluation of promising biomarkers and technologies [15]. Liquid chromatography-mass spectrometry (LC/MS) is a widely used technique for the discovery of sensitive and specific biomarkers associated with cancer [254]. This technology enables quantitative analysis of proteins using either label-based or label-free approaches [262, 266]. The label-free and label-based (using isobaric labeling, such as isobaric tags for relative and absolute quantification reagents, (iTRAQ) and tandem mass tag reagents, (TMT)) approaches have been widely used in cancer biomarker discovery and validation [267]. For example, a recent label-free quantitative proteomics study by Gautam et al., (2022) identified 16 protein biomarkers, including C-reactive protein, Carbonic anhydrase-1, and Fibronectin as putative biomarkers of oral squamous cell carcinoma (OSCC) [268]. Moulder et al., (2017) and Westbrook et al., (2014) presented many candidate protein biomarkers discovered in multiple diseases using isobaric labeling approaches [269, 270]. Proteomics-based technology can identify key information like protein targets and signaling pathways related to drug resistance, growth and metastasis of cancer cells [262].

MS-based proteomics have been applied to study many cancer types, including prostate [271, 272], breast [273–275], melanoma [276, 277], lung [278–280], ovarian [281, 282], and oropharyngeal carcinoma [283]. Furthermore, dedicated oncoproteomic reviews have been published for several malignancies, including colorectal cancer [44], breast cancer [284], prostate cancer [285], head and neck cancer [286], and lung cancer [287]. Clinical proteomic studies compare the proteomic profiles from cancerous tissue samples with “healthy” controls from the same patient or between patients with varying stages of cancer to identify potential diagnostic and prognostic biomarkers, respectively. In both cases, a number of differentially expressed candidate proteins will be identified and pathway analysis give insight in to how these proteins are associated with tumorigenesis, proliferation, metastasis and other cancer-driving processes [28].

In one of the pioneering clinical proteomic studies, Petricoin et al.,(2002) used surface-enhanced laser desorption-ionization time-of-flight mass spectrometry (SELDI-TOF MS) for diagnosing ovarian cancer [288]. OVA1 was the first clinically approved biomarker discovered using SELDI-TOF MS in 2009 [289]. Macklin et al., (2020) highlighted several clinical proteomic studies in different cancer phenotypes [28]. A comparative proteome analysis of breast tumors arising from BRCA1-deficient mouse models and -proficient triple-negative breast cancer (TNBC) identified differentially regulated nuclear protein complexes involved in homologous recombination (HR)-dependent DNA repair pathways and chromatin remodeling [290]. The proteome changes were indicative for a rescue mechanism for the loss of HR repair. This study clearly illustrates how in-depth proteomics coupled to analysis of protein functions and networks can yield a potential diagnostic and prognostic signature in BRCA1-deficient breast tumors [1, 290]. Liu et al., (2014) performed one of the largest and most comprehensive clinical proteomics studies and identified a prognostic signature that foretell diseases recurrence in TNBC patients, with high sensitivity, specificity, and positive predictive value [1, 291].

Several proteomics studies have also been performed to uncover the metastatic potential seen in cancer. A study by Obradović et al., (2019) revealed an increase in the levels stress hormone during breast cancer progression that causes an increased activity of the glucocorticoid receptor (GR) at distant metastatic sites, and ultimately reducing the survival rate [292]. Elevated expression levels of the kinase ROR1 is one of the multiple metastatic processes activated by the increased GR activity. Depletion of ROR1 resulted in reduced metastasis and extended the survival rate in preclinical models, which is in support to this study [262]. Similarly, Lignitto et al., (2019) reported an increased expression of BACH1, a pro-metastatic transcription factor, in lung adenocarcinoma [293].

Treatment resistance and development of specific and effective molecular targeted therapies are still the challenges in cancer treatment [262]. Proteomic markers are also shown to be used in guiding the selection of appropriate cancer drugs and drug targets, paving the way towards personalized medicine [294]. A study by Large et al., (2019) showed microtubule-associated protein 2 (MAP2) to be a potential biomarker for gemcitabine resistance in two cohorts of pancreatic ductal adenocarcinoma (PDAC) patients [295]. Furthermore, the group found that gemcitabine-resistant PDAC cells are sensitive to taxane-based treatment. Studies have shown that different cancer cells that are resistant to anti-cancer agents exhibit a unique protein expression and molecular mechanisms correlated to the poor survival rate of patients (revised by Kwon et al., (2021)) [262].

Proteomics has also been employed in the diagnosis of brain cancer. A study by Gupta et al., (2019) revealed that the transcription factor YBX1 was overexpressed in glioblastoma (GBM, WHO Grade IV), a potential regulator involved in tumor metastasis [296]. Another proteomic study reported that the level of CDH18, a novel tumor-suppressor, and its downstream targets were downregulated in patients with glioma than in healthy tissue [297]. A recent study demonstrated that two proteins, chitinase-3-like protein 1 (CHI3L1) and glial fibrillary acidic protein (GFAP), to be a potential CSF Biomarkers for glioma patients [298]. Kalinina et al., (2011) presented a comprehensive list of important findings on glioma proteomics [299]. Furthermore, Kwon et al., (2021) presented list of representative proteomic biomarkers against different cancer types including liver, pancreas, ovary, breast, lung, myeloid leukemia [262].

Target verification and validation are the major hurdles for the translation of potential biomarkers identified from oncoproteomic data it to the clinic [44]. Although hundreds of potential cancer biomarker candidates can be found in literature, only a limited amount of these ‘interesting’ biomarker candidates are approved by the Food and Drug Administration (FDA), as revised by Maes et al., (2015), and ultimately translated into a clinical test [14, 300, 301]. For example, FDA approved human epididymis protein 4 (HE4) in 2009, a highly sensitive and specific marker for epithelial ovarian cancer as compared to CA-125, the ‘gold standard’ ovarian cancer detection [302]. HE4 is found to be overexpressed in a number of tumours and currently used to monitor the recurrence and progression of epithelial ovarian cancer [261]. Most of the FDA-approved tumor markers are blood-based markers and complement on the regular imaging modalities in discriminating between malignant and benign states [303]. Most of the currently available cancer screenings tests usually lack sensitivity and/or specificity and the quest to find protein biomarkers able to perform early cancer diagnosis, is still ongoing [304]. Most of the blood-based tumor markers are helpful for disease staging and monitoring as they are only efficient to detect late-stage tumors in patients with an established disease to monitor disease recurrence or reduction [300]. However, most of the biomarkers are not cancer-type specific [305]. For example, elevated levels of carcinoembryonic antigen (CEA) in blood is not specific to for CRC nor for malignancy and abnormal levels of CEA expression is also demonstrated in other cancer types and also in different inflammatory diseases [306]. Advanced blood-based protein markers rely on ‘panels of multiple biomarkers’ rather than single proteins for improved diagnostic accuracy [307, 308]. Besides, FDA has approved blood-based protein markers in tissues and other non-invasive matrices, such as urine or feces [14]. The findings are more often validated by antibody-based techniques in a larger independent cohort or implemented in clinical trials.

#### Mass spectrometry-based proteomics in the diagnosis of inherited metabolic disorders

Metabolic diseases are those that result from deficiency of enzymatic activities in the catabolism of amino acids, carbohydrates, or lipids [93, 309]. The method is a powerful tool to enhance newborn screening for more than 50 different metabolic disorders in one rapid test, as opposed to the conventional enzyme- or immunoassays, which required one test to detect one disorder [26, 310]. Furthermore, MS-based method have been reported to be a cost-effective approach for newborn screening [311]. Impairment at the level of protein synthesis, stability, degradation, and signaling, all of which can play crucial roles in disease development, can be studied using proteomics technologies [312]. Expression proteomics, structural proteomics, and functional proteomics are promising approaches in the search for diagnostic biomarkers and therapeutic targets in these diseases [313, 314]. In the last decade, SWATH-MS has emerged as powerful analytical systems designed for simultaneous detection and quantification of proteins differentially expressed between healthy controls and multiple inherited metabolic disorders [315]. An organ-specific disease sampling can provide a more reliable source of potential diagnostic and therapeutic biomarkers than serum since organ-specific proteins released from diseased tissue are often diluted or degraded once they enter systemic circulation [316–318].

Recent studies have shown the potential of LC-MS/MS-based proteomics approaches in the investigation of several rare genetic metabolic disorders including lysosomal storage diseases, peroxisomal disorder, amino acid metabolism, and inborn errors of metabolism, as summarized in Chantada-Vázquez et al., (2022) [312]. Several studies have been conducted to look for different biomarker categories for lysosomal storage diseases [319]. For example, SELDI-TOF based proteomics revealed apolipoprotein ApoCI to be differentially expressed in mucopolysaccharidosis (MPS) patients compared to controls. Proteomics has also been used to analyze mutations directly at the protein level, as shown in Gaucher disease [319]. Furthermore, chitotriosidase protein (ChT) has been used as a biomarker for monitoring Gaucher disease [320]. Despite several advancements in proteomic technologies, only limited studies are performed to explore the different biomarker categories associated to inborn errors of metabolism. In conclusion, the availability of detection technology including the MS is likely to significantly improve existing newborn screening tests will prove to be beneficial for the future generations.

## Conclusion and future perspective

Although MS is still underutilized in various clinical settings, it is becoming a method of choice increasingly implemented in clinical laboratories with its multiplexing capacity, remarkable sensitivity and specificity, and potential for real-time in vivo analysis, which is often not produced by other analytical techniques [26, 46].

It is widely used in reference methods development, therapeutic drug monitoring, toxicology, endocrinology, pediatrics, immunology and microbiology to identify and quantify biomolecules in a variety of biological specimens. This new era of various screening programs, new treatments, and detection technology will prove to be beneficial for the future generations [310]. Furthermore, the pros and cons of these methods should be compared with traditional methods. Other desirable and practical features to be considered include high capital acquisition costs, requirement of skilled personnel, lack of automation, lack of direct bidirectional interface between MS instruments and laboratory information system, lack of standardization, and regulatory requirements [46, 321]. Operational factors such as standardized workflow, turnaround time, and comprehensive bio-computational data analysis and storage should also be considered.

The manufacturers and clinical MS community pursued significant progress in regard to regulatory requirements [322, 323], standardization of methods [324, 325], automation in instrumentation and data analysis [326, 327], and flat file interface to laboratory information systems to facilitate seamless order-to-result workflows [328]. Despite the remarkable promise, one of the major limitations of the clinical MS to date is the identification biomarkers at a very early stage. So far, most of clinical proteomics studies have been conducted when the disease is well manifested [26]. Over all, future technological and instrumentation advancements, development of software packages and machine learning algorithms will propel novel clinical applications of MS to the forefront [26, 46].

## Expert opinion

The continual improvement and development of new ionization methods, instrumentation and techniques, and bioinformatics tools led to the emergence of novel applications of MS-based proteomics. These instrumental and methodological advances have revolutionized the application of MS-based proteomics in the clinical research, in the context of early disease diagnosis and characterization, drug development, biomarker discovery, and predicting prognosis and drug resistance analysis.

Proteomics based method is preferred over conventional methods mainly for its multiplexing capacity, remarkable analytical sensitivity and specificity, relatively shorter turnaround time, low sample cost and potential for real-time in vivo analysis. Furthermore, the emergence of improved sample preparation and protein quantification techniques combined with the appropriate data analysis pipeline involving machine learning algorithms will identify diseases specific signatures. These achievements will hasten the transition of MS to becoming a standard component of routine analysis and are expected to become the mainstay of clinical practice.

MSI is one of the emerging advancements in the field widely used for untargeted investigation of spatial distribution of biomolecules in different samples. The acquisition of images with high spatial resolution may reduce the pixel-by-pixel sampling speed, thereby increasing the overall image acquisition and analysis time. This strategy is implemented in clinical diagnostics, biomarker discovery and drug development. Another area where MS-based proteomics could play a role is in clinical microbiology laboratory, as a tool for both infectious diseases research and diagnostic purposes. In particular, its multiplexing capacity, specificity, sensitivity and low turnaround time will improve the quality of both clinical and epidemiological data. The method is crucial in for identification and typing of emerging microbial pathogens, antibiotic resistance analysis, exploring host-pathogen interaction and pathogenesis and formulating accurate treatment plans in time thereby controlling infectious diseases effectively.

The practices in oncology clinic have now been transformed through continuous discovery and validation of cancer biomarker, which are critically important in early diagnosis, risk stratification, and monitoring patient response to treatment. Oncoproteomics is more likely to reflect the comprehensive changes implicated in tumorigenesis than genomics and transcriptomics. This approach is already opening new avenues for the identification of novel biomarkers for early detection, targeted therapies, disease monitoring, and drug development, thereby advancing the implementation of personalized medicine.

Metabolic diseases are among the most serious medical problems that modern societies face. It includes a broad spectrum of biochemical alterations caused by cellular (genetic) defects and/or environmental factors which affects the structure and function of proteins involved in cellular metabolic pathways, thereby contribute to the pathogenesis of metabolic diseases. In this context, proteomic analysis has emerged as an indispensable tool to elucidate the complex molecular basis of various pathophysiological processes and protein dynamics through identification, quantification and structure characterizations of hundreds of proteins from a single complex biological sample. Metabolites and proteins are attractive diagnostic and therapeutic biomarkers in metabolic diseases since their concentration (deficiency or accumulation) is implicated in disease pathways.

The size and mobility of the MS systems are also the challenges in the transition of MS technique in on-site scenarios, such as ambulances and outdoors for POC diagnosis. The emergence new equipment like the miniature MS systems fill the gap by direct sampling in their native environment without pretreatment, thereby reducing turnaround time and the roles of skilled operators (mainly in sample preparation). Further progress in these areas will continue to provide researchers with new insights and technologies that will benefit the general population. The technology is rapidly advancing and could be amenable to automation, user-friendly protocols that would translate well to the clinic. The advantages and limitations MS-based clinical proteomics over traditional methods need to be established and communicated among clinical laboratory technicians, researchers, and other potential users of these novel methods.

For the proper clinical implementation of all these achievements, operational factors and other desirable practical features such as automation, standardized protocols and software packages, machine learning algorithms, comprehensive bio-computational data analysis and storage should also be considered. These recent developments with others will advance the translation of MS from analytical chemistry to clinical labs to provide POC service and personalized treatment for patients in future. Furthermore, as these instrumentation and technologies become more automated, accessible and affordable with certain MS platforms already well-entrenched in research or clinical laboratories, clinical proteomics has a great future ahead for improving disease diagnosis, prognosis, monitoring and prediction of therapeutic outcome.

## Data Availability

Not applicable.

## References

[1] Jimenez CR, Verheul HM (2014). Mass spectrometry-based proteomics: from cancer biology to protein biomarkers, drug targets, and clinical applications. Am Soc Clin Oncol Educational Book.

[2] Sevimoglu T, Arga KY (2014). The role of protein interaction networks in systems biomedicine. Comput Struct Biotechnol J.

[3] Gonzalez MW, Kann MG. Chap. 4: *Protein interactions and disease* PLoS computational biology, 2012. 8(12): p. e1002819. 10.1371/journal.pcbi.1002819 PMC3531279 23300410

[4] Khodadadi E (2020). Proteomic applications in antimicrobial resistance and clinical microbiology studies. Infect Drug Resist.

[5] Iwamoto N, Shimada T. Recent advances in mass spectrometry-based approaches for proteomics and biologics: great contribution for developing therapeutic antibodies. Volume 185. Pharmacology & Therapeutics; 2018. pp. 147–54. 10.1016/j.pharmthera.2017.12.007 29274706

[6] Clarke NJ, Zhang Y, Reitz RE (2012). A novel mass spectrometry–based assay for the accurate measurement of thyroglobulin from patient samples containing antithyroglobulin autoantibodies. J Investig Med.

[7] Rostaing L (2010). Falsely elevated whole-blood tacrolimus concentrations in a kidney‐transplant patient: potential hazards. Transpl Int.

[8] Hanash S (2003). Disease proteomics. Nature.

[9] Oliveira BM (2013). Is clinical proteomics heading towards to “bench to bedside. Transl Proteom.

[10] Mischak H (2012). How to get proteomics to the clinic? Issues in clinical proteomics, exemplified by CE-MS. PROTEOMICS–Clinical Appl.

[11] Mischak H (2012). Implementation of proteomic biomarkers: making it work. Eur J Clin Invest.

[12] Baker ES (2012). Mass spectrometry for translational proteomics: progress and clinical implications. Genome Med.

[13] Boja ES, Rodriguez H (2011). The path to clinical proteomics research: integration of proteomics, genomics, clinical laboratory and regulatory science. Korean J Lab Med.

[14] Maes E (2015). Proteomics in cancer research: are we ready for clinical practice?. Crit Rev Oncol/Hematol.

[15] Findeisen P, Neumaier M (2009). Mass spectrometry-based clinical proteomics profiling: current status and future directions. Expert Rev Proteomics.

[16] Lill JR (2021). Proteomics in the pharmaceutical and biotechnology industry: a look to the next decade. Expert Rev Proteomics.

[17] Parker CE, Borchers CH (2014). Mass spectrometry based biomarker discovery, verification, and validation–quality assurance and control of protein biomarker assays. Mol Oncol.

[18] Srinivas PR (2002). Proteomics for cancer biomarker discovery. Clin Chem.

[19] Olsson B (2016). CSF and blood biomarkers for the diagnosis of Alzheimer’s disease: a systematic review and meta-analysis. Lancet Neurol.

[20] Jang HN et al. *Mass Spectrometry-Based Proteomic Discovery of prognostic biomarkers in adrenal cortical carcinoma*. Cancers (Basel), 2021. 13(15). 10.3390/cancers13153890 PMC8345732 34359790

[21] Gam L-H (2012). Breast cancer and protein biomarkers. World J experimental Med.

[22] Hamdan MH. Cancer biomarkers: analytical techniques for discovery. John Wiley & Sons; 2007.

[23] Amiri-Dashatan N (2018). Proteomics applications in health: biomarker and drug discovery and food industry. Iran J Pharm research: IJPR.

[24] Yang WS (2012). Proteomic approach reveals FKBP4 and S100A9 as potential prediction markers of therapeutic response to neoadjuvant chemotherapy in patients with breast cancer. J Proteome Res.

[25] Zhang D, Putti TC (2010). Over-expression of ERp29 attenuates doxorubicin-induced cell apoptosis through up-regulation of Hsp27 in breast cancer cells. Exp Cell Res.

[26] Banerjee S (2020). Empowering clinical diagnostics with mass spectrometry. ACS omega.

[27] Blattmann P, Aebersold R. *The Advent of Mass Spectrometry-Based Proteomics in Systems Biology Research* 2016.

[28] Macklin A, Khan S, Kislinger T (2020). Recent advances in mass spectrometry based clinical proteomics: applications to cancer research. Clin Proteom.

[29] Lange V (2008). Selected reaction monitoring for quantitative proteomics: a tutorial. Mol Syst Biol.

[30] Wu W, Dai R-T, Bendixen E (2019). Comparing SRM and SWATH methods for quantitation of bovine muscle proteomes. J Agric Food Chem.

[31] Mermelekas G, Vlahou A, Zoidakis J (2015). SRM/MRM targeted proteomics as a tool for biomarker validation and absolute quantification in human urine. Expert Rev Mol Diagn.

[32] Jones JJ (2016). A plasma-based protein marker panel for Colorectal Cancer Detection identified by Multiplex targeted Mass Spectrometry. Clin Colorectal Cancer.

[33] Kontostathi G (2019). Applications of multiple reaction monitoring targeted proteomics assays in human plasma. Expert Rev Mol Diagn.

[34] Boys EL (2023). Clinical applications of mass spectrometry-based proteomics in cancer: where are we?. Proteomics.

[35] Ludwig C (2018). Data-independent acquisition‐based SWATH‐MS for quantitative proteomics: a tutorial. Mol Syst Biol.

[36] Tully B (2019). Addressing the challenges of high-throughput cancer tissue proteomics for clinical application: proCan. Proteomics.

[37] Poulos RC (2020). Strategies to enable large-scale proteomics for reproducible research. Nat Commun.

[38] Gillet LC, et al. Targeted data extraction of the MS/MS spectra generated by data-independent acquisition: a new concept for consistent and accurate proteome analysis. Volume 11. Molecular & Cellular Proteomics; 2012. 6. 10.1074/mcp.O111.016717 PMC3433915 22261725

[39] Collins BC (2017). Multi-laboratory assessment of reproducibility, qualitative and quantitative performance of SWATH-mass spectrometry. Nat Commun.

[40] Reubsaet L, Sweredoski MJ, Moradian A (2018). Data-independent acquisition for the Orbitrap Q Exactive HF: a tutorial. J Proteome Res.

[41] Chang RY (2015). SWATH analysis of the synaptic proteome in Alzheimer’s disease. Neurochem Int.

[42] Kim YJ (2018). Targeted data-independent acquisition for mass spectrometric detection of RAS mutations in formalin-fixed, paraffin-embedded tumor biopsies. J Proteom.

[43] Anjo SI, Santa C, Manadas B (2017). SWATH-MS as a tool for biomarker discovery: from basic research to clinical applications. Proteomics.

[44] de Wit M (2013). Proteomics in colorectal cancer translational research: biomarker discovery for clinical applications. Clin Biochem.

[45] Patterson SD, Aebersold RH (2003). Proteomics: the first decade and beyond. Nat Genet.

[46] Fung AW (2020). Emerging role of clinical mass spectrometry in pathology. J Clin Pathol.

[47] Satoh M, Nomura F (2019). Applications of mass spectrometry in clinical chemistry. Med Mass Spectrom.

[48] Stone J. Sample preparation techniques for mass spectrometry in the clinical laboratory, Mass Spectrometry for the Clinical Laboratory. 2017, Elsevier. 37–62.

[49] Principe S (2012). Identification of prostate-enriched proteins by in-depth proteomic analyses of expressed prostatic secretions in urine. J Proteome Res.

[50] Drake RR (2010). In-depth proteomic analyses of direct expressed prostatic secretions. J Proteome Res.

[51] Wu CC (2015). Saliva proteome profiling reveals potential salivary biomarkers for detection of oral cavity squamous cell carcinoma. Proteomics.

[52] de Souza GA, de Godoy LM, Mann M (2006). Identification of 491 proteins in the tear fluid proteome reveals a large number of proteases and protease inhibitors. Genome Biol.

[53] Spreafico F (2017). Proteomic analysis of cerebrospinal fluid from children with central nervous system tumors identifies candidate proteins relating to tumor metastatic spread. Oncotarget.

[54] Elschenbroich S (2011). In-depth proteomics of ovarian cancer ascites: combining shotgun proteomics and selected reaction monitoring mass spectrometry. J Proteome Res.

[55] Gortzak-Uzan L (2008). A proteome resource of ovarian cancer ascites: integrated proteomic and bioinformatic analyses to identify putative biomarkers. J Proteome Res.

[56] Ding Z (2022). Proteomics technologies for cancer liquid biopsies. Mol Cancer.

[57] Anderson NL (2004). Mass spectrometric quantitation of peptides and proteins using stable isotope Standards and capture by Anti-Peptide antibodies (SISCAPA). J Proteome Res.

[58] Denti V (2020). Antigen Retrieval and its effect on the MALDI-MSI of lipids in Formalin-Fixed paraffin-embedded tissue. J Am Soc Mass Spectrom.

[59] Ly A (2016). High-mass-resolution MALDI mass spectrometry imaging of metabolites from formalin-fixed paraffin-embedded tissue. Nat Protoc.

[60] Buczak K (2020). Spatially resolved analysis of FFPE tissue proteomes by quantitative mass spectrometry. Nat Protoc.

[61] Eckert MA (2019). Proteomics reveals NNMT as a master metabolic regulator of cancer-associated fibroblasts. Nature.

[62] Sinha A (2019). The proteogenomic landscape of curable prostate cancer. Cancer Cell.

[63] Wang H (2005). Development and evaluation of a micro-and nanoscale proteomic sample preparation method. J Proteome Res.

[64] Frantzi M (2016). Development and validation of urine-based peptide biomarker panels for detecting bladder Cancer in a multi-center StudyDevelopment of urinary biomarker panels for bladder Cancer. Clin Cancer Res.

[65] Lee H-J, Kim H-J, Liebler DC (2016). Efficient microscale basic reverse phase peptide fractionation for global and targeted proteomics. J Proteome Res.

[66] Cooper HJ (2016). To what extent is FAIMS beneficial in the analysis of proteins?. J Am Soc Mass Spectrom.

[67] Michelmann K (2014). Fundamentals of trapped ion mobility spectrometry. J Am Soc Mass Spectrom.

[68] Hebert AS (2018). Comprehensive single-shot proteomics with FAIMS on a hybrid orbitrap mass spectrometer. Anal Chem.

[69] Manza LL (2005). Sample preparation and digestion for proteomic analyses using spin filters. Proteomics.

[70] Wiśniewski JR (2009). Universal sample preparation method for proteome analysis. Nat Methods.

[71] Berger ST, et al. MStern blotting–high throughput polyvinylidene fluoride (PVDF) membrane-based Proteomic Sample Preparation for 96-Well Plates*[S]. Volume 14. Molecular & Cellular Proteomics; 2015. pp. 2814–23. 10. 10.1074/mcp.O115.049650 PMC4597154 26223766

[72] Zougman A, Selby PJ, Banks RE (2014). Suspension trapping (STrap) sample preparation method for bottom-up proteomics analysis. Proteomics.

[73] HaileMariam M (2018). S-Trap, an ultrafast sample-preparation approach for shotgun proteomics. J Proteome Res.

[74] Hughes CS (2014). Ultrasensitive proteome analysis using paramagnetic bead technology. Mol Syst Biol.

[75] Leutert M (2019). R2-P2 rapid‐robotic phosphoproteomics enables multidimensional cell signaling studies. Mol Syst Biol.

[76] Hughes CS (2016). Quantitative profiling of single formalin fixed tumour sections: proteomics for translational research. Sci Rep.

[77] Owen DR (2018). Molecular characterization of ERBB2-amplified colorectal cancer identifies potential mechanisms of resistance to targeted therapies: a report of two instructive cases. Mol Case Stud.

[78] Kulak NA (2014). Minimal, encapsulated proteomic-sample processing applied to copy-number estimation in eukaryotic cells. Nat Methods.

[79] Ludwig KR, Schroll MM, Hummon AB (2018). Comparison of in-solution, FASP, and S-trap based digestion methods for bottom-up proteomic studies. J Proteome Res.

[80] Sielaff M (2017). Evaluation of FASP, SP3, and iST protocols for proteomic sample preparation in the low microgram range. J Proteome Res.

[81] Hughes CS (2019). Single-pot, solid-phase-enhanced sample preparation for proteomics experiments. Nat Protoc.

[82] Li C et al. *Towards higher sensitivity of mass spectrometry: a perspective from the mass analyzers*. Front Chem, 2021. 9. 10.3389/fchem.2021.813359 PMC8724130 34993180

[83] Makarov A (2019). Orbitrap journey: taming the ion rings. Nat Commun.

[84] Andrews GL (2011). Performance characteristics of a new hybrid quadrupole time-of-flight tandem mass spectrometer (TripleTOF 5600). Anal Chem.

[85] Schilling B (2015). Multiplexed, scheduled, high-resolution parallel reaction monitoring on a full scan QqTOF instrument with integrated data-dependent and targeted mass spectrometric workflows. Anal Chem.

[86] Nyadong L (2013). Laserspray and matrix-assisted ionization inlet coupled to high-field FT-ICR mass spectrometry for peptide and protein analysis. J Am Soc Mass Spectrom.

[87] Hendrickson CL (2015). 21 Tesla Fourier transform ion cyclotron resonance mass spectrometer: a national resource for ultrahigh resolution mass analysis. J Am Soc Mass Spectrom.

[88] Jarmusch AK (2016). Lipid and metabolite profiles of human brain tumors by desorption electrospray ionization-MS. Proc Natl Acad Sci.

[89] Yoshimura K (2015). Development of non-proximate probe electrospray ionization for real-time analysis of living animal. Mass Spectrom.

[90] St John ER (2017). Rapid evaporative ionisation mass spectrometry of electrosurgical vapours for the identification of breast pathology: towards an intelligent knife for breast cancer surgery. Breast Cancer Res.

[91] Sinitcyn P (2021). MaxDIA enables library-based and library-free data-independent acquisition proteomics. Nat Biotechnol.

[92] Chen C (2020). Bioinformatics methods for mass spectrometry-based proteomics data analysis. Int J Mol Sci.

[93] Taunk K (2020). The development and clinical applications of proteomics: an indian perspective. Expert Rev Proteomics.

[94] Chong Y-K (2018). Clinical mass spectrometry in the bioinformatics era: a Hitchhiker’s guide. Comput Struct Biotechnol J.

[95] Anderson L (2005). Candidate-based proteomics in the search for biomarkers of cardiovascular disease. J Physiol.

[96] Paulovich AG (2008). The interface between biomarker discovery and clinical validation: the tar pit of the protein biomarker pipeline. Proteom Clin Appl.

[97] Whiteaker JR (2007). Integrated pipeline for mass spectrometry-based discovery and confirmation of biomarkers demonstrated in a mouse model of breast cancer. J Proteome Res.

[98] Kiyonami R et al. *Increased selectivity, analytical precision, and throughput in targeted proteomics*. Mol Cell Proteomics, 2011. 10(2): p. M110.002931. 10.1074/mcp.M110.002931 PMC3033677 20664071

[99] Addona TA (2009). Multi-site assessment of the precision and reproducibility of multiple reaction monitoring-based measurements of proteins in plasma. Nat Biotechnol.

[100] Anderson NL (2010). The clinical plasma proteome: a survey of clinical assays for proteins in plasma and serum. Clin Chem.

[101] van der Gugten JG (2020). Tandem mass spectrometry in the clinical laboratory: a tutorial overview. Clin Mass Spectrom.

[102] DeMarco ML (2020). An automated clinical mass spectrometric method for identification and quantification of variant and wild-type amyloid-β 1–40 and 1–42 peptides in CSF. Alzheimers Dement (Amst).

[103] Chambers AG (2014). MRM for the verification of cancer biomarker proteins: recent applications to human plasma and serum. Expert Rev Proteomics.

[104] Foster AG (2013). Rapid identification of microbes in positive blood cultures by use of the Vitek MS matrix-assisted laser desorption ionization–time of flight mass spectrometry system. J Clin Microbiol.

[105] Faron ML (2015). Multicenter evaluation of the Bruker MALDI Biotyper CA System for the identification of clinical aerobic gram-negative bacterial isolates. PLoS ONE.

[106] Frank KM, McManus LM, Mitchell RN (2014). *Microbiology in Clinical Pathology*, in *Pathobiology of Human Disease*.

[107] Nolen BM, Lokshin AE (2013). Biomarker testing for ovarian cancer: clinical utility of multiplex assays. Mol Diagn Ther.

[108] Boja ES (2014). Analytical Validation Considerations of Multiplex Mass-Spectrometry-Based proteomic platforms for measuring protein biomarkers. J Proteome Res.

[109] Clarke W, Rhea JM, Molinaro R (2013). Challenges in implementing clinical liquid chromatography-tandem mass spectrometry methods–the light at the end of the tunnel. J Mass Spectrom.

[110] Turck CW (2007). The association of biomolecular resource facilities proteomics research group 2006 study: relative protein quantitation. Mol Cell Proteom.

[111] Kim SR (2015). Comparative proteomics: assessment of biological variability and dataset comparability. BMC Bioinformatics.

[112] Bag AK (2014). Comparative proteomics and glycoproteomics of plasma proteins in indian visceral leishmaniasis. Proteome Sci.

[113] Minden J (2007). Comparative proteomics and difference gel electrophoresis. Biotechniques.

[114] Volmer DA, Mendes LR, Stokes CS (2015). Analysis of vitamin D metabolic markers by mass spectrometry: current techniques, limitations of the “gold standard” method, and anticipated future directions. Mass Spectrom Rev.

[115] Nguyen CDL (2019). A sensitive and simple targeted proteomics approach to quantify transcription factor and membrane proteins of the unfolded protein response pathway in glioblastoma cells. Sci Rep.

[116] Ronsein GE (2015). Parallel reaction monitoring (PRM) and selected reaction monitoring (SRM) exhibit comparable linearity, dynamic range and precision for targeted quantitative HDL proteomics. J Proteom.

[117] Zhou X, Zhang W, Ouyang Z. *Recent advances in on-site mass spectrometry analysis for clinical applications*. TRAC Trends Anal Chem, 2022: p. 116548. 10.1016/j.trac.2022.116548 PMC8802081 35125564

[118] Chamberlain CA, Rubio VY, Garrett TJ (2019). Strain-level differentiation of Bacteria by Paper Spray Ionization Mass Spectrometry. Anal Chem.

[119] Longuespée R (2016). MALDI mass spectrometry imaging: a cutting-edge tool for fundamental and clinical histopathology. PROTEOMICS–Clinical Appl.

[120] Kriegsmann J, Kriegsmann M, Casadonte R (2015). MALDI TOF imaging mass spectrometry in clinical pathology: a valuable tool for cancer diagnostics. Int J Oncol.

[121] Jannetto PJ, Fitzgerald RL (2016). Effective use of mass spectrometry in the clinical laboratory. Clin Chem.

[122] Buchberger AR (2018). Mass spectrometry imaging: a review of emerging advancements and future insights. Anal Chem.

[123] Banerjee S (2018). Ambient ionization mass spectrometry imaging for disease diagnosis: excitements and challenges. J Biosci.

[124] Eberlin LS et al. *Desorption electrospray ionization mass spectrometry for lipid characterization and biological tissue imaging* Biochimica et Biophysica Acta (BBA)-Molecular and Cell Biology of Lipids, 2011. 1811(11): p. 946–60. 10.1016/j.bbalip.2011.05.006 PMC3205348 21645635

[125] Barry JA (2014). Mapping antiretroviral drugs in tissue by IR-MALDESI MSI coupled to the Q exactive and comparison with LC-MS/MS SRM assay. J Am Soc Mass Spectrom.

[126] Yang H (2020). Mass Spectrometry Imaging of Microbes. Mass Spectrom Lett.

[127] Arentz G (2017). Applications of mass spectrometry imaging to cancer. Adv Cancer Res.

[128] Ucal Y (2017). Clinical applications of MALDI imaging technologies in cancer and neurodegenerative diseases. Biochim et Biophys Acta (BBA)-Proteins Proteom.

[129] Eberlin LS (2010). Discrimination of human astrocytoma subtypes by lipid analysis using desorption electrospray ionization imaging mass spectrometry. Angew Chem.

[130] Ifa DR, Eberlin LS (2016). Ambient ionization mass spectrometry for cancer diagnosis and surgical margin evaluation. Clin Chem.

[131] Banerjee S, Manna SK. Assessment of metabolic signature for Cancer diagnosis using Desorption Electrospray Ionization Mass Spectrometric Imaging, Cancer Metabolism. 2019, Springer. 275–97. 10.1007/978-1-4939-9027-6_15 30725461

[132] Holzlechner M, Eugenin E, Prideaux B (2019). Mass spectrometry imaging to detect lipid biomarkers and disease signatures in cancer. Cancer Rep.

[133] Kriegsmann J (2018). Mass spectrometry in pathology–vision for a future workflow. Pathology-Research and Practice.

[134] Diehl HC (2015). The challenge of on-tissue digestion for MALDI MSI—a comparison of different protocols to improve imaging experiments. Anal Bioanal Chem.

[135] Hermann J (2020). Sample preparation of formalin-fixed paraffin-embedded tissue sections for MALDI-mass spectrometry imaging. Anal Bioanal Chem.

[136] Leung F (2019). Mass spectrometry-based tissue imaging: the next frontier in clinical diagnostics?. Clin Chem.

[137] Perez CJ (2019). Review and perspectives on the applications of mass spectrometry imaging under ambient conditions. Rapid Commun Mass Spectrom.

[138] Schäfer KC (2009). In vivo, in situ tissue analysis using rapid evaporative ionization mass spectrometry. Angew Chem Int Ed.

[139] Golf O (2015). Rapid evaporative ionization mass spectrometry imaging platform for direct mapping from bulk tissue and bacterial growth media. Anal Chem.

[140] Balog J (2013). Intraoperative tissue identification using rapid evaporative ionization mass spectrometry. Sci Transl Med.

[141] Balog J (2010). Identification of biological tissues by rapid evaporative ionization mass spectrometry. Anal Chem.

[142] Zhang J (2017). Nondestructive tissue analysis for ex vivo and in vivo cancer diagnosis using a handheld mass spectrometry system. Sci Transl Med.

[143] Alexander J (2017). A novel methodology for in vivo endoscopic phenotyping of colorectal cancer based on real-time analysis of the mucosal lipidome: a prospective observational study of the iKnife. Surg Endosc.

[144] Greco TM, Cristea IM (2017). Proteomics tracing the footsteps of infectious disease. Mol Cell Proteom.

[145] Greco TM, Diner BA, Cristea IM (2014). The impact of mass spectrometry–based proteomics on fundamental discoveries in virology. Annual Rev Virol.

[146] Wu F, Zhong F, He F (2016). Microbial proteomics: approaches, advances, and applications. J Bioinf Proteom Imaging Anal.

[147] Jean Beltran PM (2017). Proteomics and integrative omic approaches for understanding host–pathogen interactions and infectious diseases. Mol Syst Biol.

[148] Singhal N (2015). MALDI-TOF mass spectrometry: an emerging technology for microbial identification and diagnosis. Front Microbiol.

[149] Sauer S, Kliem M (2010). Mass spectrometry tools for the classification and identification of bacteria. Nat Rev Microbiol.

[150] Welker M, Moore ER (2011). Applications of whole-cell matrix-assisted laser-desorption/ionization time-of-flight mass spectrometry in systematic microbiology. Syst Appl Microbiol.

[151] Idelevich EA, Becker K (2021). Matrix-assisted laser desorption ionization–time of Flight Mass Spectrometry for Antimicrobial susceptibility testing. J Clin Microbiol.

[152] Clark AE (2013). Matrix-assisted laser desorption ionization–time of flight mass spectrometry: a fundamental shift in the routine practice of clinical microbiology. Clin Microbiol Rev.

[153] Lum KK, Cristea IM (2016). Proteomic approaches to uncovering virus–host protein interactions during the progression of viral infection. Expert Rev Proteomics.

[154] Lee C-R (2015). Quantitative proteomic view associated with resistance to clinically important antibiotics in Gram-positive bacteria: a systematic review. Front Microbiol.

[155] Stekhoven DJ (2014). Proteome-wide identification of predominant subcellular protein localizations in a bacterial model organism. J Proteom.

[156] Sperk M (2020). Utility of proteomics in emerging and re-emerging infectious diseases caused by RNA viruses. J Proteome Res.

[157] Havlicek V, Lemr K, Schug KA (2013). Current trends in microbial diagnostics based on mass spectrometry. Anal Chem.

[158] Seng P (2010). MALDI-TOF-mass spectrometry applications in clinical microbiology. Future Microbiol.

[159] Holland R (1996). Rapid identification of intact whole bacteria based on spectral patterns using matrix-assisted laser desorption/ionization with time‐of‐flight mass spectrometry. Rapid Commun Mass Spectrom.

[160] Cox CR, Voorhees KJ. Bacterial identification by Mass Spectrometry, Detection of Chemical, Biological, Radiological and Nuclear Agents for the Prevention of Terrorism. 2014, Springer. 115–31.

[161] Bizzini A (2010). Performance of matrix-assisted laser desorption ionization-time of flight mass spectrometry for identification of bacterial strains routinely isolated in a clinical microbiology laboratory. J Clin Microbiol.

[162] Steensels D, Verhaegen J, Lagrou K (2011). Matrix-assisted laser desorption ionization-time of flight mass spectrometry for the identification of bacteria and yeasts in a clinical microbiological laboratory: a review. Acta Clin Belg.

[163] Berendsen EM (2017). Identification of microorganisms grown in blood culture flasks using liquid chromatography–tandem mass spectrometry. Future Microbiol.

[164] Lasch P (2020). Identification of microorganisms by liquid chromatography-mass spectrometry (LC-MS1) and in silico peptide mass libraries. Mol Cell Proteom.

[165] Kostrzewa M (2018). Application of the MALDI biotyper to clinical microbiology: progress and potential. Expert Rev Proteomics.

[166] Bahk YY (2004). Antigens secreted from Mycobacterium tuberculosis: identification by proteomics approach and test for diagnostic marker. Proteomics.

[167] La Scola B, Raoult D (2009). Direct identification of bacteria in positive blood culture bottles by matrix-assisted laser desorption ionisation time-of-flight mass spectrometry. PLoS ONE.

[168] Stevenson LG, Drake SK, Murray PR (2010). Rapid identification of bacteria in positive blood culture broths by matrix-assisted laser desorption ionization-time of flight mass spectrometry. J Clin Microbiol.

[169] Haigh J (2013). Rapid identification of bacteria from bioMerieux BacT/ALERT blood culture bottles by MALDI-TOF MS. Br J Biomed Sci.

[170] Tadros M, Petrich A (2013). Evaluation of MALDI-TOF mass spectrometry and Sepsityper Kit™ for the direct identification of organisms from sterile body fluids in a canadian pediatric hospital. Can J Infect Dis Med Microbiol.

[171] Nomura F (2020). Mass spectrometry-based microbiological testing for blood stream infection. Clin Proteomics.

[172] Guembe M et al. *Can MALDI-TOF mass spectrometry be used with intravascular catheters?* Enfermedades Infecciosas y Microbiología Clínica, 2014. 32(6): p. 372–4. 10.1016/j.eimc.2014.01.011 24679820

[173] Roux-Dalvai F, et al. Fast and accurate bacterial species identification in urine specimens using LC-MS/MS Mass Spectrometry and Machine Learning*[S]. Volume 18. Molecular & Cellular Proteomics; 2019. pp. 2492–505. 12. 10.1074/mcp.TIR119.001559 PMC6885708 31585987

[174] Kitagawa K (2018). Improved bacterial identification directly from urine samples with matrix-assisted laser desorption/ionization time‐of‐flight mass spectrometry. J Clin Lab Anal.

[175] Ferreira L (2010). Direct identification of urinary tract pathogens from urine samples by matrix-assisted laser desorption ionization-time of flight mass spectrometry. J Clin Microbiol.

[176] Köhling HL (2012). Direct identification of bacteria in urine samples by matrix-assisted laser desorption/ionization time-of-flight mass spectrometry and relevance of defensins as interfering factors. J Med Microbiol.

[177] Burillo A (2014). Gram-stain plus MALDI-TOF MS (matrix-assisted laser desorption ionization-time of flight mass spectrometry) for a rapid diagnosis of urinary tract infection. PLoS ONE.

[178] El-Sadek WAE-L (2021). Matrix-assisted laser desorption ionization-time of Flight Mass Spectrometry (MALDI-TOF MS) for the identification of Bacteria causing urinary tract infections. Egypt J Med Microbiol.

[179] Pinault L (2019). Direct identification of pathogens in urine by use of a specific matrix-assisted laser desorption ionization–time of flight spectrum database. J Clin Microbiol.

[180] He Y (2010). Mass spectrometry biotyper system identifies enteric bacterial pathogens directly from colonies grown on selective stool culture media. J Clin Microbiol.

[181] Dierig A, Frei R, Egli A. *The fast route to microbe identification: matrix assisted laser desorption/ionization—time of flight mass spectrometry (MALDI-TOF MS)* The Pediatric infectious disease journal, 2015. 34(1): p. 97–9. 10.1097/INF.0000000000000601 25741802

[182] Segawa S (2014). Direct application of MALDI-TOF mass spectrometry to cerebrospinal fluid for rapid pathogen identification in a patient with bacterial meningitis. Clin Chim Acta.

[183] Tsuchida S, Umemura H, Nakayama T (2020). Current status of matrix-assisted laser desorption/ionization–time-of-flight mass spectrometry (MALDI-TOF MS) in clinical diagnostic microbiology. Molecules.

[184] Bishop B (2018). The use of matrix-assisted laser desorption/ionization time-of-flight mass spectrometry for rapid bacterial identification in patients with smear-positive bacterial meningitis. Clin Microbiol Infect.

[185] Luo Y (2015). Performance of the VITEK MS matrix-assisted laser desorption ionization-time of flight mass spectrometry system for rapid bacterial identification in two diagnostic centres in China. J Med Microbiol.

[186] Pecora N, Milner DA Jr. New Technologies for the diagnosis of infection. Diagnostic Pathology of Infectious Disease; 2018. pp. 104–17.

[187] Patel R (2015). MALDI-TOF MS for the diagnosis of infectious diseases. Clin Chem.

[188] Yoon E-J, Jeong SH (2021). MALDI-TOF mass spectrometry technology as a tool for the rapid diagnosis of antimicrobial resistance in bacteria. Antibiotics.

[189] Florio W (2020). Detection of antibiotic-resistance by MALDI-TOF mass spectrometry: an expanding area. Front Cell Infect Microbiol.

[190] Aleshukina A (2022). Mass spectrometric study of antibiotic resistance of S. aureus and P. aeruginosa using the MALDIquant package. Int J Infect Dis.

[191] Weis C (2022). Direct antimicrobial resistance prediction from clinical MALDI-TOF mass spectra using machine learning. Nat Med.

[192] Charretier Y, Schrenzel J (2016). Mass spectrometry methods for predicting antibiotic resistance. PROTEOMICS–Clinical Appl.

[193] Kliem M, Sauer S (2012). The essence on mass spectrometry based microbial diagnostics. Curr Opin Microbiol.

[194] Sjöholm MI, Dillner J, Carlson J (2008). Multiplex detection of human herpesviruses from archival specimens by using matrix-assisted laser desorption ionization-time of flight mass spectrometry. J Clin Microbiol.

[195] Yi X (2011). A new PCR-based mass spectrometry system for high-risk HPV, part I: methods. Am J Clin Pathol.

[196] Du H (2011). A new PCR-based mass spectrometry system for high-risk HPV, part II: clinical trial. Am J Clin Pathol.

[197] Piao J et al. *Simultaneous detection and identification of enteric viruses by PCR-mass assay* 2012. 10.1371/journal.pone.0042251 PMC3411642 22870310

[198] Downard KM, Morrissey B, Schwahn AB (2009). Mass spectrometry analysis of the influenza virus. Mass Spectrom Rev.

[199] Ren Y (2004). The use of proteomics in the discovery of serum biomarkers from patients with severe acute respiratory syndrome. Proteomics.

[200] Luan J (2009). Multiplex detection of 60 hepatitis B virus variants by maldi-tof mass spectrometry. Clin Chem.

[201] Peng J (2013). Sensitive and rapid detection of viruses associated with hand foot and mouth disease using multiplexed MALDI-TOF analysis. J Clin Virol.

[202] Calderaro A (2014). Matrix-assisted laser desorption/ionization time-of-flight (MALDI-TOF) mass spectrometry applied to virus identification. Sci Rep.

[203] Calderaro A (2016). Identification of different respiratory viruses, after a cell culture step, by matrix assisted laser desorption/ionization time of flight mass spectrometry (MALDI-TOF MS). Sci Rep.

[204] Ihling C (2020). Mass spectrometric identification of SARS-CoV-2 proteins from gargle solution samples of COVID-19 patients. J Proteome Res.

[205] Yan L (2021). Rapid detection of COVID-19 using MALDI-TOF-based serum peptidome profiling. Anal Chem.

[206] Nachtigall FM (2020). Detection of SARS-CoV-2 in nasal swabs using MALDI-MS. Nat Biotechnol.

[207] Roberts DS (2021). Structural O-glycoform heterogeneity of the SARS-CoV-2 spike protein receptor-binding domain revealed by top-down mass spectrometry. J Am Chem Soc.

[208] De Silva IW (2020). Paper spray mass spectrometry utilizing Teslin® substrate for rapid detection of lipid metabolite changes during COVID-19 infection. Analyst.

[209] Cardozo KHM (2020). Establishing a mass spectrometry-based system for rapid detection of SARS-CoV-2 in large clinical sample cohorts. Nat Commun.

[210] Van Puyvelde B (2021). Cov-MS: a community-based template assay for mass-spectrometry-based protein detection in SARS-CoV-2 patients. Jacs Au.

[211] Van Puyvelde B et al. *Cov2MS: an automated matrix-independent assay for mass spectrometric detection and measurement of SARS-CoV-2 nucleocapsid protein in infectious patients*. medRxiv, 2022. 10.1021/acs.analchem.2c01610 PMC9773173 36490367

[212] Hober A (2021). Rapid and sensitive detection of SARS-CoV-2 infection using quantitative peptide enrichment LC-MS analysis. Elife.

[213] Maarten D. *Add mass spectrometry to the pandemic toolbox*. eLife, 2021. 10. 10.7554/eLife.75471 PMC8664293 34889738

[214] Kavallaris M, Marshall GM (2005). Proteomics and disease: opportunities and challenges. Med J Aust.

[215] Ying W (2004). Proteomic analysis on structural proteins of severe Acute Respiratory Syndrome coronavirus. Proteomics.

[216] Zürcher S (2012). Sensitive and rapid detection of ganciclovir resistance by PCR based MALDI-TOF analysis. J Clin Virol.

[217] Sendid B (2013). Evaluation of MALDI-TOF mass spectrometry for the identification of medically-important yeasts in the clinical laboratories of Dijon and Lille hospitals. Med Mycol.

[218] Posteraro B (2013). MALDI-TOF mass spectrometry in the clinical mycology laboratory: identification of fungi and beyond. Expert Rev Proteomics.

[219] Santos C (2010). Filamentous fungal characterizations by matrix-assisted laser desorption/ionization time‐of‐flight mass spectrometry. J Appl Microbiol.

[220] Giebel R (2010). Microbial fingerprinting using matrix-assisted laser desorption ionization time-of-flight mass spectrometry (MALDI-TOF MS): applications and challenges. Adv Appl Microbiol.

[221] Qian J (2008). MALDI-TOF mass signatures for differentiation of yeast species, strain grouping and monitoring of morphogenesis markers. Anal Bioanal Chem.

[222] Pulcrano G (2012). MALDI-TOF mass spectrometry and microsatellite markers to evaluate Candida parapsilosis transmission in neonatal intensive care units. Eur J Clin Microbiol Infect Dis.

[223] Krüger T (2015). Challenges and strategies for proteome analysis of the interaction of human pathogenic fungi with host immune cells. Proteomes.

[224] Amiri-Eliasi B, Fenselau C (2001). Characterization of protein biomarkers desorbed by MALDI from whole fungal cells. Anal Chem.

[225] Spanu T (2012). Direct MALDI-TOF mass spectrometry assay of blood culture broths for rapid identification of Candida species causing bloodstream infections: an observational study in two large microbiology laboratories. J Clin Microbiol.

[226] Yaman G, Akyar I, Can S (2012). Evaluation of the MALDI TOF-MS method for identification of Candida strains isolated from blood cultures. Diagn Microbiol Infect Dis.

[227] Lavergne R-A (2013). An extraction method of positive blood cultures for direct identification of Candida species by Vitek MS matrix-assisted laser desorption ionization time of flight mass spectrometry. Med Mycol.

[228] Marinach-Patrice C (2010). Rapid species diagnosis for invasive candidiasis using mass spectrometry. PLoS ONE.

[229] Normand A-C (2017). Decision criteria for MALDI-TOF MS-based identification of filamentous fungi using commercial and in-house reference databases. BMC Microbiol.

[230] Cassagne C (2011). Mould routine identification in the clinical laboratory by matrix-assisted laser desorption ionization time-of-flight mass spectrometry. PLoS ONE.

[231] Lau AF (2013). Development of a clinically comprehensive database and a simple procedure for identification of molds from solid media by matrix-assisted laser desorption ionization–time of flight mass spectrometry. J Clin Microbiol.

[232] Normand A-C (2013). Assessment of various parameters to improve MALDI-TOF MS reference spectra libraries constructed for the routine identification of filamentous fungi. BMC Microbiol.

[233] Ranque S (2014). MALDI-TOF mass spectrometry identification of filamentous fungi in the clinical laboratory. Mycoses.

[234] Schulthess B (2014). Use of the Bruker MALDI biotyper for identification of molds in the clinical mycology laboratory. J Clin Microbiol.

[235] De Carolis E (2012). Species identification of aspergillus, Fusarium and Mucorales with direct surface analysis by matrix-assisted laser desorption ionization time-of-flight mass spectrometry. Clin Microbiol Infect.

[236] L’Ollivier C (2013). A MALDI-TOF MS procedure for clinical dermatophyte species identification in the routine laboratory. Med Mycol.

[237] Del Chierico F (2012). MALDI-TOF MS proteomic phenotyping of filamentous and other fungi from clinical origin. J Proteom.

[238] Nenoff P (2013). MALDI-TOF mass spectrometry–a rapid method for the identification of dermatophyte species. Med Mycol.

[239] Packeu A (2013). Identification of the Trichophyton mentagrophytes complex species using MALDI-TOF mass spectrometry. Med Mycol.

[240] Packeu A (2014). Fast and accurate identification of dermatophytes by matrix-assisted laser desorption ionization–time of flight mass spectrometry: validation in the clinical laboratory. J Clin Microbiol.

[241] Gautier M (2014). Matrix-assisted laser desorption ionization time-of-flight mass spectrometry: revolutionizing clinical laboratory diagnosis of mould infections. Clin Microbiol Infect.

[242] Bader O (2013). MALDI-TOF‐MS‐based species identification and typing approaches in medical mycology. Proteomics.

[243] Pfaller M (2002). Antifungal activities of posaconazole, ravuconazole, and voriconazole compared to those of itraconazole and amphotericin B against 239 clinical isolates of Aspergillus spp. and other filamentous fungi: report from SENTRY Antimicrobial Surveillance Program, 2000. Antimicrob Agents Chemother.

[244] Alastruey-Izquierdo A (2010). Antifungal susceptibility profile of human-pathogenic species of Lichtheimia. Antimicrob Agents Chemother.

[245] Siegel RL, Miller KD, Jemal A. *Cancer statistics*, 2018. CA Cancer J Clin, 2018. 68(1): p. 7–30. 10.3322/caac.21442 29313949

[246] Siegel RL, Miller KD, Jemal A (2020). Cancer statistics, 2020. CA Cancer J Clin.

[247] Siegel RL (2022). Cancer statistics, 2022. CA Cancer J Clin.

[248] Mertins P (2016). Proteogenomics connects somatic mutations to signalling in breast cancer. Nature.

[249] Zhang B (2014). Proteogenomic characterization of human colon and rectal cancer. Nature.

[250] Zhang H (2016). Integrated proteogenomic characterization of human high-grade serous ovarian cancer. Cell.

[251] Yaffe MB (2019). Why geneticists stole cancer research even though cancer is primarily a signaling disease. Sci Signal.

[252] Coscia F (2018). Multi-level proteomics identifies CT45 as a chemosensitivity mediator and immunotherapy target in ovarian cancer. Cell.

[253] Aebersold R (2018). How many human proteoforms are there?. Nat Chem Biol.

[254] Smith LM, Kelleher NL (2013). Proteoform: a single term describing protein complexity. Nat Methods.

[255] Corso S (2008). Silencing the MET oncogene leads to regression of experimental tumors and metastases. Oncogene.

[256] Cleary AS (2014). Tumour cell heterogeneity maintained by cooperating subclones in wnt-driven mammary cancers. Nature.

[257] Koren S, Bentires-Alj M (2015). Breast tumor heterogeneity: source of Fitness, Hurdle for Therapy. Mol Cell.

[258] Köbel M (2008). Ovarian carcinoma subtypes are different diseases: implications for biomarker studies. PLoS Med.

[259] Boussios S (2020). Veliparib in ovarian cancer: a new synthetically lethal therapeutic approach. Invest New Drugs.

[260] Lheureux S, Braunstein M, Oza AM (2019). Epithelial ovarian cancer: evolution of management in the era of precision medicine. CA Cancer J Clin.

[261] Ghose A et al. *Applications of Proteomics in Ovarian Cancer: Dawn of a new era*. Proteomes, 2022. 10(2). 10.3390/proteomes10020016 PMC9150001 35645374

[262] Kwon YW et al. *Application of proteomics in cancer: recent trends and approaches for biomarkers discovery*. Front Med, 2021. 8. 10.3389/fmed.2021.747333 PMC8492935 34631760

[263] Rudnick PA (2016). A description of the clinical proteomic Tumor Analysis Consortium (CPTAC) Common Data Analysis Pipeline. J Proteome Res.

[264] Taylor CF (2007). The minimum information about a proteomics experiment (MIAPE). Nat Biotechnol.

[265] Martínez-Bartolomé S, Binz P-A, Albar JP. *The minimal information about a proteomics experiment (MIAPE) from the proteomics standards initiative*, in *Plant Proteomics*. Springer; 2014. pp. 765–80. 10.1007/978-1-62703-631-3_53 24136562

[266] Kay R (2017). Liquid chromatography/mass spectrometry based detection and semi-quantitative analysis of INSL5 in human and murine tissues. Rapid Commun Mass Spectrom.

[267] Kumar V et al. *An Integrated quantitative proteomics Workflow for Cancer Biomarker Discovery and Validation in plasma*. Front Oncol, 2020. 10. 10.3389/fonc.2020.543997 PMC7538778 33072574

[268] Gautam SS (2022). Label-free plasma proteomics for the identification of the putative biomarkers of oral squamous cell carcinoma. J Proteom.

[269] Moulder R (2018). Analysis of the plasma proteome using iTRAQ and TMT-based isobaric labeling. Mass Spectrom Rev.

[270] Westbrook JA (2015). Quantitation with chemical tagging reagents in biomarker studies. Proteom Clin Appl.

[271] Guo T et al. *Multi-region proteome analysis quantifies spatial heterogeneity of prostate tissue biomarkers*. Life Sci alliance, 2018. 1(2). 10.26508/lsa.201800042 PMC6078179 30090875

[272] Shao W (2019). Comparative analysis of mRNA and protein degradation in prostate tissues indicates high stability of proteins. Nat Commun.

[273] Pozniak Y (2016). System-wide clinical proteomics of breast cancer reveals global remodeling of tissue homeostasis. Cell Syst.

[274] Yanovich G (2018). Clinical proteomics of breast Cancer reveals a Novel layer of breast Cancer ClassificationClinical Proteomics analysis of breast Cancer classification. Cancer Res.

[275] Tyanova S (2016). Proteomic maps of breast cancer subtypes. Nat Commun.

[276] Bassani-Sternberg M (2016). Direct identification of clinically relevant neoepitopes presented on native human melanoma tissue by mass spectrometry. Nat Commun.

[277] Harel M (2019). Proteomics of melanoma response to immunotherapy reveals mitochondrial dependence. Cell.

[278] Doll S (2018). Rapid proteomic analysis for solid tumors reveals LSD 1 as a drug target in an end-stage cancer patient. Mol Oncol.

[279] He W (2018). CTHRC1 induces non-small cell lung cancer (NSCLC) invasion through upregulating MMP-7/MMP-9. BMC Cancer.

[280] Li L (2014). Integrated omic analysis of lung cancer reveals metabolism proteome signatures with prognostic impact. Nat Commun.

[281] Li QK (2017). An integrated proteomic and glycoproteomic approach uncovers differences in glycosylation occupancy from benign and malignant epithelial ovarian tumors. Clin Proteomics.

[282] Dieters-Castator DZ (2019). Proteomics-derived Biomarker Panel improves Diagnostic Precision to classify endometrioid and high-grade Serous Ovarian CarcinomaPrecision Biomarker Set for Ovarian Cancer classification. Clin Cancer Res.

[283] Sepiashvili L (2014). Integrated omic analysis of oropharyngeal carcinomas reveals human papillomavirus (HPV)–dependent regulation of the activator protein 1 (AP-1) pathway. Mol Cell Proteom.

[284] Lam S, Jimenez C, Boven E (2014). Breast cancer classification by proteomic technologies: current state of knowledge. Cancer Treat Rev.

[285] Pin E, Fredolini C, Petricoin EF (2013). The role of proteomics in prostate cancer research: biomarker discovery and validation. Clin Biochem.

[286] Schaaij-Visser TB (2010). Protein biomarker discovery for head and neck cancer. J Proteom.

[287] Indovina P (2013). Mass spectrometry-based proteomics: the road to lung cancer biomarker discovery. Mass Spectrom Rev.

[288] Petricoin III (2002). Use of proteomic patterns in serum to identify ovarian cancer. The lancet.

[289] Zhang Z (2004). Three biomarkers identified from serum proteomic analysis for the detection of early stage ovarian cancer. Cancer Res.

[290] Warmoes M (2012). Proteomics of mouse BRCA1-deficient mammary tumors identifies DNA repair proteins with potential diagnostic and prognostic value in human breast cancer. Mol Cell Proteom.

[291] Liu NQ et al. *Comparative proteome analysis revealing an 11-protein signature for aggressive triple-negative breast cancer*. JNCI: J Natl Cancer Inst, 2014. 106(2). 10.1093/jnci/djt376 PMC3952199 24399849

[292] Obradović MMS (2019). Glucocorticoids promote breast cancer metastasis. Nature.

[293] Lignitto L (2019). Nrf2 activation promotes Lung Cancer Metastasis by inhibiting the degradation of Bach1. Cell.

[294] An Y (2019). Molecular insights into cancer drug resistance from a proteomics perspective. Expert Rev Proteomics.

[295] Le Large TYS (2019). Proteomic analysis of gemcitabine-resistant pancreatic cancer cells reveals that microtubule-associated protein 2 upregulation associates with taxane treatment. Ther Adv Med Oncol.

[296] Gupta MK (2019). Altered transcriptional regulatory proteins in glioblastoma and YBX1 as a potential regulator of tumor invasion. Sci Rep.

[297] Bai YH (2018). A novel Tumor-Suppressor, CDH18, inhibits Glioma Cell Invasiveness Via UQCRC2 and correlates with the prognosis of Glioma Patients. Cell Physiol Biochem.

[298] Schmid D (2021). Diagnostic biomarkers from proteomic characterization of cerebrospinal fluid in patients with brain malignancies. J Neurochem.

[299] Kalinina J (2011). Proteomics of gliomas: initial biomarker discovery and evolution of technology. Neuro Oncol.

[300] Hanash SM (2011). Why have protein biomarkers not reached the clinic?. Genome Med.

[301] Füzéry AK (2013). Translation of proteomic biomarkers into FDA approved cancer diagnostics: issues and challenges. Clin Proteomics.

[302] Montagnana M (2011). HE4 in ovarian cancer: from discovery to clinical application. Adv Clin Chem.

[303] Hanash SM, Baik CS, Kallioniemi O (2011). Emerging molecular biomarkers—blood-based strategies to detect and monitor cancer. Nat reviews Clin Oncol.

[304] Diamandis EP (2014). Towards identification of true cancer biomarkers. BMC Med.

[305] Diamandis EP (2012). The failure of protein cancer biomarkers to reach the clinic: why, and what can be done to address the problem?. BMC Med.

[306] Yamashita K, Watanabe M (2009). Clinical significance of tumor markers and an emerging perspective on colorectal cancer. Cancer Sci.

[307] Nolen B (2010). Serum biomarker panels for the discrimination of benign from malignant cases in patients with an adnexal mass. Gynecol Oncol.

[308] Leung F (2013). Advances in mass spectrometry-based technologies to direct personalized medicine in ovarian cancer. Transl Proteom.

[309] Costanzo M (2017). Integration of Proteomics and Metabolomics in Exploring Genetic and Rare Metabolic Diseases. Kidney Dis (Basel).

[310] Yoon H-R (2015). Screening newborns for metabolic disorders based on targeted metabolomics using tandem mass spectrometry. Annals of Pediatric Endocrinology & Metabolism.

[311] Yu M (2022). Cost-effectiveness analysis of newborn screening by tandem mass spectrometry in Shenzhen, China: value and affordability of new screening technology. BMC Health Serv Res.

[312] Chantada-Vázquez MDP et al. *Proteomics in inherited metabolic Disorders*. Int J Mol Sci, 2022. 23(23). 10.3390/ijms232314744 PMC9740208 36499071

[313] Aslam B (2017). Proteomics: Technologies and their applications. J Chromatogr Sci.

[314] Richard E (2009). Proteomics as Applied to inherited metabolic Diseases. Curr Proteom.

[315] Gillet LC, et al. Targeted data extraction of the MS/MS Spectra generated by Data-independent Acquisition: a New Concept for consistent and Accurate Proteome Analysis*. Volume 11. Molecular & Cellular Proteomics; 2012. 6O111.016717. 10.1074/mcp.O111.016717 PMC3433915 22261725

[316] Dayon L, Cominetti O, Affolter M (2022). Proteomics of human biological fluids for biomarker discoveries: technical advances and recent applications. Expert Rev Proteomics.

[317] Geyer PE (2017). Revisiting biomarker discovery by plasma proteomics. Mol Syst Biol.

[318] Lone SN (2022). Liquid biopsy: a step closer to transform diagnosis, prognosis and future of cancer treatments. Mol Cancer.

[319] Richard E (2009). Proteomics as Applied to inherited metabolic Diseases. Curr Proteomics.

[320] Hollak CE (1994). Marked elevation of plasma chitotriosidase activity. A novel hallmark of Gaucher disease. J Clin Invest.

[321] Crutchfield CA (2016). Advances in mass spectrometry-based clinical biomarker discovery. Clin Proteomics.

[322] Lynch KL (2016). CLSI C62-A: a new standard for clinical mass spectrometry. Clin Chem.

[323] Lynch KL (2018). Accreditation and quality assurance for clinical liquid Chromatography–Mass spectrometry laboratories. Clin Lab Med.

[324] Vesper HW, Botelho JC (2010). Standardization of testosterone measurements in humans. J Steroid Biochem Mol Biol.

[325] Wise SA (2017). Baseline assessment of 25-hydroxyvitamin D assay performance: a vitamin D standardization program (VDSP) interlaboratory comparison study. J AOAC Int.

[326] Dickerson JA (2013). Design and implementation of software for automated quality control and data analysis for a complex LC/MS/MS assay for urine opiates and metabolites. Clin Chim Acta.

[327] Vicente FB, Lin DC, Haymond S (2019). Automation of chromatographic peak review and order to result data transfer in a clinical mass spectrometry laboratory. Clin Chim Acta.

[328] Holmes DT. *Flat File Interface your Mass Spectrometer to the Laboratory Information System with R* 2016 https://www.r-bloggers.com/2016/02/flat-file-interface-your-mass-spectrometer-to-the-laboratory-information-system-with-r/.

